# USP29‐regulated noncanonical stabilization of the hypoxia‐inducible factor‐α in aggressive prostate cancer

**DOI:** 10.1002/1878-0261.70268

**Published:** 2026-05-13

**Authors:** Amelie S Schober, Leire Moreno‐Cugnon, Laura Martínez‐Pérez, Amaia Ercilla, Amaia Zabala‐Letona, Adrián Vicente‐Barrueco, Maider Fagoaga‐Eugui, Saioa Garcia‐Longarte, Blanca Calle‐Ciborro, Encarnación Pérez‐Andrés, Onintza Carlevaris, Sara Pozo, Isabel Mendizabal, Ugo Mayor, Arkaitz Carracedo, Violaine Sée, Edurne Berra

**Affiliations:** ^1^ Centro de Investigación Cooperativa en Biociencias CIC bioGUNE, Basque Research and Technology Alliance (BRTA) Parque Tecnológico de Bizkaia‐Ed.801A Derio Spain; ^2^ Institute of Integrative Biology, Department of Biochemistry, Centre for Cell Imaging University of Liverpool UK; ^3^ Centro de Investigación Biomédica en Red de Cáncer (CIBERONC) Madrid Spain; ^4^ Ikerbasque, Basque Foundation for Science Bilbao Bizkaia Spain; ^5^ Translational Prostate Cancer Research Lab, CIC bioGUNE‐Basurto Biocruces Bizkaia Research Health Institute Barakaldo Spain; ^6^ Department of Biochemistry and Molecular Biology University of the Basque Country (UPV/EHU) Leioa Bizkaia Spain; ^7^ Present address: CNRS UMR 5305, Tissue Biology and Therapeutic Engineering Laboratory (LBTI), Universite Claude Bernard Lyon1 France

**Keywords:** deubiquitinases, HIF, hypoxia, prostate cancer, USP29

## Abstract

Oxygen availability is frequently compromised in solid tumours, making intratumoural hypoxia a common feature of cancer. In prostate cancer (PCa), hypoxia is strongly associated with aggressive disease and poor prognosis. Hypoxia‐inducible factor (HIF) is the master transcriptional regulator mediating hypoxia adaptation and is mainly controlled through proteasomal degradation of its α‐subunit by the ubiquitin–proteasome system (UPS). However, the contribution of deubiquitinases (DUBs) to HIF signalling in PCa remains largely unexplored. Using a computational strategy based on CA9 expression as a surrogate of HIF activity, we identified Ubiquitin‐Specific Protease 29 (USP29) as a key regulator associated with hypoxia and tumour progression and severity in PCa. Mechanistically, USP29 functions as a noncanonical positive regulator of HIF‐α stability in a catalytic‐dependent manner. USP29 interacts with HIF‐1α, reduces its poly‐ubiquitination and protects it from proteasomal degradation across multiple cancer cell lines. Additionally, USP29 stabilizes HIF‐2α acting on the C‐terminal region of HIF‐α. These findings uncover a novel regulatory layer of HIF signalling and highlight USP29 as a potential therapeutic target in hypoxia‐driven PCa progression.

## Graphical abstract

We identify USP29 as the only DUB mirroring CA9 expression, a marker of hypoxia and HIF pathway activation associated with PCA aggressiveness. USP29 stabilizes HIF‐1α and HIF‐2α via a noncanonical mechanism that is independent of PHD/pVHL activity yet relies on proteasomal regulation, establishing USP29 as a previously unrecognized regulator of hypoxic responses in PCA.

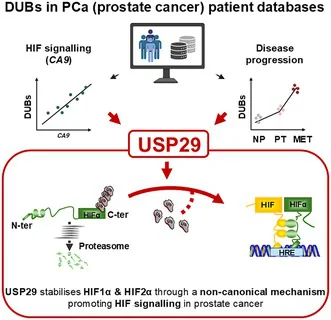

**Abbreviations**
CA9: carbonic anhydrase 9
CHX: cycloheximide
DMOG: dimethyloxalylglycine
DOX: doxycycline
DUBs: deubiquitinating enzymes
EGLN: egl nine homologues
EMT: epithelial‐to‐mesenchymal transition
FLIM‐FRET: fluorescence lifetime imaging microscopy‐föster/fluorescence resonance energy transfer
GS: Gleason score
H: human
HIF: hypoxia‐inducible factor
HRE: hypoxia responsive elements
Hx: hypoxia
M: mouse
MET: metastatic tumours
MG132: Z‐L‐Leu‐D‐Leu‐L‐Leu‐al
NEM: N‐ethylmaleimide
NP: nontumoural tissue
Nx: normoxia
PCa: prostate cancer
PHD: prolyl hydroxylase domain proteins.
PT: primary tumours
R: rat
SDS/PAGE: sodium dodecyl sulphate‐polyacrylamide gel electrophoresis
SNAI2: zinc finger SNAI2
T: cow
Ub: ubiquitin
UPS: ubiquitin–proteasome system
USP29: Ubiquitin‐Specific Protease 29
VHL: Von‐Hippel–Lindau
WCE: whole‐cell extracts
X: xenopus
Z: zebrafish
ZEB2: zinc finger E‐box‐binding homeobox 2

## 1 Introduction

Hypoxia is present in 90% of solid tumours and is strongly associated with poor prognosis in various cancer types, including prostate cancer (PCa), which is among the most frequent cancer in men [1, 2]. PCa is predominantly diagnosed at a localized stage and treated with largely effective first‐line therapies [3]. However, a subset of patients will exhibit disease recurrence months to years after treatment. Despite second‐line treatments, the emergence of metastasis in these patients is frequent, representing the major risk of mortality by PCa. Prostate cancer cells and their environment co‐opt the hypoxia signalling pathway to drive cancer progression [4, 5]. Hypoxia‐inducible transcription factors (HIFs) orchestrate the cellular adaptation to the hypoxic tumour microenvironment [6]. HIF is a dimer whose activation primarily relies on the oxygen‐dependent regulation of its alpha subunit stability [7, 8]. HIF activation triggers the transcription of hundreds of direct target genes, indirect transcription factors and noncoding RNAs that contribute to every critical aspect of cancer progression, including angiogenesis, cancer stem cell specification, cell motility, epithelial‐mesenchymal transition, extracellular matrix remodelling, metabolic rewiring, immune evasion, invasion, resistance to chemo‐ and radiotherapy and metastasis [9]. Accordingly, extended HIF activation and HIF‐α staining in tumours have been associated with higher aggressiveness, migratory and metastasis‐initiating potential and, therefore, worse prognosis [10, 11].

Hypoxia signalling and HIF‐α protein stability are tightly regulated through the dynamic interplay between ubiquitination and deubiquitination via the ubiquitin–proteasome system (UPS) [8]. In well‐oxygenated cells, HIF‐α is canonically hydroxylated by the oxygen sensors PHDs/EGLNs (Egl nine homologues), subsequently ubiquitinated by the ubiquitin E3‐ligase complex containing von‐Hippel–Lindau protein (pVHL) and finally degraded by the proteasome [12, 13, 14, 15, 16]. Upon hypoxia, PHDs/EGLNs' activity is compromised, and HIF‐α escapes from degradation, dimerizes with HIF‐β, binds to RCGTG motifs (hypoxia responsive elements, HRE) within the regulatory domains of target genes, and transcriptionally drives their expression [17].

The family of deubiquitinating enzymes (DUBs) is key in the UPS through its ability to specifically deconjugate ubiquitin (Ub) moieties from targeted proteins [18]. The key role of DUBs is the removal of ubiquitin chains from target proteins, opposing the action of the Ub E3 ligases, which are responsible for target protein ubiquitination. The coaction of the two sets of enzymes allows the cell to adapt its overall protein content, localization and function to a variety of cellular and environmental stresses, including hypoxia and HIF signalling. Dysfunction, mutations or altered expression of more than 30 DUBs contribute directly or indirectly to many disorders, including cancer [19]. DUBs act as both tumour suppressors and oncogenes. Therefore, DUBs represent an emerging therapeutic paradigm in cancer. Indeed, DUBs, like proteases, are pharmaceutical targets thanks to their well‐characterized catalytical domain [20]. The human genome encodes up to 100 DUBs, divided into seven subgroups based on their sequence and structure [19]. Despite the notable advances made over the past two decades, there are still critical gaps in uncovering the link between DUBs and hypoxia signalling networks [21, 22, 23, 24, 25].

Here, we hypothesized that DUBs implicated in HIF‐dependent signalling could contribute to PCa development and metastasis. This could reveal new therapeutic targets for treating PCa and help improve patient outcomes, which remain poor despite substantial efforts to advance current therapies [3]. We performed a bioinformatic analysis to identify DUBs that regulate HIF signalling in PCa. Using publicly available transcriptomic data from PCa patients, we identified USP29, also known as HOM‐TEST‐84/86, as one of the TOP‐10 candidates whose expression positively correlates with the HIF‐target *CA9*. In line with the role of hypoxia and CAIX expression as drivers of tumour progression, USP29 expression is associated with PCa aggressiveness. We further demonstrated that this DUB is an activator of hypoxia signalling by regulating HIF‐1α and HIF‐2α stability through a noncanonical (PHDs/pVHL‐independent) yet proteasome‐dependent mechanism.

## 2 Materials and methods

### 2.1 Plasmids

HIF‐1α^630‐826^ was amplified via PCR from pCMV‐Myc‐HIF‐1α and inserted into the BamHI/ApaI‐digested pCMV‐Myc‐HIF‐1α vector. Green fluorescent protein‐tagged HIF‐1α DM^(P402/564A)^ was generated by inserting the sequence of Clover (no.: 40259; Addgene plasmid) behind the Myc‐tag in the BamHI‐digested pCMV‐Myc‐HIF‐1α DM^(PP/AA)^ construct [26] using In‐Fusion HD Cloning (Clontech). Then, replacing HIF‐1α DM^(PP/AA)^ with HIF‐2α DM^(PP/AA)^ green fluorescent, HIF‐2α DM^(PP/AA)^ was generated. mCherry‐USP29 was cloned by inserting the PCR‐amplified mCherry sequence [27] between the BspEI and NheI restriction sites of GFP‐USP29. HIF‐α truncations (HIF‐1α^630‐713^, HIF‐1α^630‐750^, HIF‐1α^1‐657^ and HIF‐2α^601‐870^) as well as HIF‐1α DM^K752/755/758R(KKK/RRR)^, HIF‐2α DM^(P405/531A)^, HA‐USP29 siRNA‐resistant and the catalytically inactive USP29^C294S^ were generated using the QuikChange^®^ II XL Site‐Directed Mutagenesis Kit (Stratagene). Sub‐cloning of shRNAs into pLKO‐Tet‐On vector (no.: 21915; Addgene plasmid) was done by introducing *AgeI* and *EcoRI* in the 5´end of top and bottom shRNA oligos, respectively. HA‐USP29 was amplified by PCR and inserted into the AgeI/MluI‐digested doxycycline‐inducible lentiviral TRIPZ vector (Dharmacon) to generate the TRIPZ‐USP29 construct. Table 1 recapitulates the sequences of all the oligos used for cloning and mutagenesis. All the constructs were verified by sequencing. HIF‐2α‐Myc has been described before [27]. The FLAG‐ubiquitin plasmids were a gift from [28]. HA‐USP29 and GFP‐USP29 expression vectors were gifts from [29].

**Table 1:** Oligo sequences for cloning and mutagenesis of HIF‐α and USP29 plasmids.

PCR amplification	Oligos (5′–3′)
HIF‐1α^630‐826^	F: ATGGGATCCGACCGTATGGAAGA R: CATGGGCCCTCAGTTAACTTGATCC
Clover for HIF‐1α DM^(PP/AA)^	F: GATCTGAGCCCGGGCGGAGTGAGCAAGGGCGAGGAGCTG R: GGAATTCCGGGGATCCCTTGTACAGCTCGTCCATGCCATG
HIF‐2α DM^(PP/AA)^	F: ACGAGCTGTACAAGGGAACAGCTGACAAGGAGAAGAAAAG R: TTAATTAAGGTACCGCGGTGGCCTGGTCCAGGGC
mCherry for USP29	F: GAACCGTCAGATCCGCCACCATGGTGAGCAAGGGCGAG R: CTCGAGATCTGAGTCCGGACTTGTACAGCTCGTCCATGCCGC
pLKO‐Tet‐On‐shControl	F: CCGGCATCATCGATCGGGGATGTAGGCTCGAGCCTACATCCCCGATCGATGATGTTTTT R: AATTAAAAA CATCATCGATCGGGGATGTAGGCTCGAGCCTACATCCCCGATCGATGATG
pLKO‐Tet‐On‐shUSP29.1	F: CCGGTTTCCAGATTTGAAAGTGACCACTCGAGTGGTCACTTTCAAATCTGGAAATTTTT R: AATTAAAAATTTCCAGATTTGAAAGTGACCACTCGAGATGGTCACTTTCAAATCTGGAAA
pLKO‐Tet‐On‐shUSP29.2	F: CCGGTTTCCAGATTTGAAAGTGACCACTCGAGTGGTCACTTTCAAATCTGGAAA TTTTT R: AATTAAAAATTTCCAGATTTGAAAGTGACCACTCGAGTGGTCACTTTCAAATCTGGAAA
TRIPZ‐USP29	F: AACCGTCAGATCGCAACCATGGCTTATCCTTACGACGTGCCTGACTACGCCATATC TCTAAAGGTATGTGGATTCA R: CGCGGAGGCCACGCGTCAAGCAGGTCTGTACAAAGAGTCA

Mutagenesis	Oligos (5′–3′)
HIF‐1α^630‐713^	F: CCAAAGATACTAGCTTTGTAGAATGCTCAGAGAAAGCGAAAAATG R: CATTTTTCGCTTTCTCTGAGCATTCTACAAAGCTAGTATCTTTGG
HIF‐1α^630‐750^	F: CATGCAGCTACTACATCACTTTGATGGAAACGTGTAAAAGGATG R: CATCCTTTTACACGTTTCCATCAAAGTGATGTAGTAGCTGCATG
HIF‐1α^K752/755/758R^	F: CATCACTTTCTTGGAGACGTGTAAGAGGATGTAGATCTAGTGAACAG R: CTGTTCACTAGATCTACATCCTCTTACACGTCTCCAAGAAAGTGATG
HIF‐2α^P405A^	F: CAATGAGCTGGACCTCGAGACACTGGCAGCCTATATCCCCATG R: CATGGGGATATAGGCTGCCAGTGTCTCGAGGTCCAGCTCATTG
HIF‐2α^P531A^	F: GAGGAGCTGGCCCAGCTAGCTGCCACCCCAGGAGACGCC R: GGCGTCTCCTGGGGTGGCAGCTAGCTGGGCCAGCTCCTC
USP29^C294S^	F: CCCCAATTTGGGAAACACCAGTTACATGAATGCAGTTTTAC R: GTAAAACTGCATTCATGTAACTGGTGTTTCCCAAATTGGGG
USP29 siRNA‐resistant	F: GAAAGCAGGAATATGCTCAAAGAGATTGACAAAACTTCATTTTACGC R: GCGTAAAATGAAGTTTTGTCAATCTCTTTGAGCATATTCCTGCTTTC

### 2.2 Cell culture and transfections

Human HEK 293 T (RRID: CVCL_0063), HeLa (RRID: CVCL_0030), SK‐N‐As (RRID: CVCL_1700), SH‐SY5Y (RRID: CVCL_0019) and MDA‐MB‐231 (RRID: CVCL_0062) cell lines were purchased from American Type Culture Collection, which provided an authentication certificate. Human PCa PC3 (RRID:CVCL_E2RM) and LNCaP (RRID:CVCL_0395) cells were purchased at Leibniz Institut DSMZ (Deutsche Sammlung con Mikroorganismen und Zelkulturen GmbH), with the corresponding certificate of authenticity. Human ovarian cancer cells A2780 (RID:CVCL_0134) were a kind gift provided by the laboratory of Dr. Francesc Vinals. Cell lines were subjected to microsatellite‐based identity validation, and low‐passage validated cell lines were stored in liquid nitrogen. For the experiments in this study, validated cells were thawed and employed. All the cell lines were monitored for mycoplasma contamination, and experiments were performed in mycoplasma‐free cells. HEK293T cells were cultured in Dulbecco's modified Eagle medium (DMEM) supplemented with 5% FBS. HeLa and PC3 cells were cultured in DMEM supplemented with 10% FBS and SK‐N‐AS cells with 1% nonessential amino acids additionally. A2780 and LNCaP were cultured in RPMI supplemented with 10% FBS, and MDA‐MB‐231 and SH‐SY5Y cells were cultured in DMEM:F12 (1 : 1) supplemented with 10% FBS. Cells were incubated at 37°C at 95% humidity and 5% CO_2_.

siRNAs and shRNAs were purchased from SIGMA (Table 2). For transient expression of siRNAs or DNAs, cells were transfected in suspension at plating or 24 h postseeding at 60–70% confluence, respectively, using Lipofectamine 2000 (Invitrogen) as a transfection reagent following the manufacturer's instructions. Forty‐eight hours after seeding and the first transfection, cells were ready for experimental treatment and/or harvested. Lentiviral transductions were performed as previously described [30]. Selection was done using puromycin (2 μg·mL^−1^) for 3 days.

**Table 2:** Sequences of shRNAs and siRNAs.

	Sequence (5′–3′)
shControl	CATCATCGATCGGGGATGTAGG
shHIF1A	TCCTGTGGTGACTTGTCCTT
shUSP29.1 (v2HS_200524)	TTGATCTCAGAAATCATCTCCT
shUSP29.2 (v2HS_250889)	TTTCCAGATTTGAAAGTGACCA
siControl	CCUACAUCCCGAUCGAUGAUGdTdT
siUSP29.1	GGAAUAUGCUGAAGGAAAUdTdT
siUSP29.2	GGUCACUUUCAAAUCUGGAdTdT
siPHD2	CUUCAGAUUCGGUCGGUAAAGdTdT
sipVHL	GGAGCGCAUUGCACAUCAACGdTdT

Incubation in hypoxia was achieved in an anaerobic workstation (*In vivo*_2_ 400; Ruskinn), and cell lysis was performed inside the anaerobic workstation to avoid reoxygenation.

### 2.3 Proliferation and colony formation assay by crystal violet staining

PC3 (pLKO‐Tet‐On and TRIPZ) cells were seeded in media with or without doxycycline (0.5 μg·mL^−1^). Subclonfluent cells (5.000 cells per well in a 6‐well plate on triplicate) were cultured for 1, 3 or 6 days for proliferation assay. For the colony formation assay, 250 cells per well were seeded in a 6‐well plate in triplicate. The cells were allowed to grow and form foci for up to 14 days. Cells were finally washed using 1× PBS, fixed with formalin and further stained with crystal violet as described in [30].

### 2.4 Cell cycle analysis

PC3 cells (5000 cells per well on a 96‐well microplate) were seeded in triplicate. Ten micrometres of EdU was added 30 min before collecting the plates and fixing them in 4% formaldehyde for 15 min at room temperature (RT). Fixed cells were permeabilized by incubating the cells in 0.2% Triton X‐100 PBS for 30 min at RT. Click reaction was performed by incubating the cells in Click‐it buffer [100 mm Tris–HCl pH 8, 2 mm CuSO_4_, 0.5 ng·mL^−1^ Alexa Fluor 488 Azide (A10266; Life Technologies), and 100 mm ascorbic acid] for 30 min at RT. Cells were incubated in 0.5 μg·mL^−1^ DAPI containing PBS for 30 min at RT. Fresh filtered PBS was added to the microplates for imaging. Images were obtained automatically with the ScanR acquisition software controlling a motorized Olympus IX‐83 wide‐field microscope. Images were processed using the scanr image analysis software. tibco spotfire^®^ software was used to analyse the data.

### 2.5 Wound‐healing assays

Wound‐healing assays were performed by seeding 200.000 cells in 6‐well plates at high confluency. PC3 (pLKO‐Tet‐On and TRIPZ) cells were incubated with or without doxycycline (0.5 μg·mL^−1^) for 3 days in the presence or the absence of DMOG (1 mm) overnight. A 20 μL pipet tip was used to wound the cell monolayer. Images were acquired at 0, 8 and 24 h using an Olympus IX‐83 inverted microscope operated by the CellSens software. Cell migration rate was measured by calculating the linear growth of wound closure and dividing the slope by 2 times the length of the initial area of the scratch. Experiments were performed in triplicate. fiji software was used to quantify the wounded area.

### 2.6 RNA isolation and RT‐qPCR

Total RNA was automatically extracted using Maxwell^®^ RSC instrument and Maxwell RSC simplyRNA Cells Kit (Promega) according to the manufacturers' instructions. RNA concentration was quantified by ThermoScientific Nanodrop One spectrophotometer before being retro‐transcribed into cDNA using MaximaTM H Minus cDNA Synthesis Master Mix. Quantitative PCR (qPCR) was performed using cDNA as a template and TaqMan Universal Master Mix II in combination with primers and Universal Probe Library (UPL) probes (*ZEB2*: FWD 5′cgatccagaccgcaattaac3′, REV 5′tgctgactgcatgaccatc3′, UPL#65; *SNAI2*: FWD 5′tggttgcttcaaggacacat3′, REV 5′gcaaatgctctgttgcagtg3′, UPL#7). Reactions were conducted on QS5 Quant Studio Real‐Time PCR systems (Life Technologies), and the relative expression of each target mRNA was calculated using the 2^−ΔΔCt^ method with *RPLP0* serving as the reference gene for normalization.

### 2.7 Animals

Mouse experiments were conducted in accordance with ethical guidelines established by the Biosafety and Animal Welfare Committee at CIC bioGUNE (Spanish acronym for centre for cooperative research in Biosciences) following recommendations from the Association for Assessment and Accreditation of Laboratory Animal Care International (AAALAC), and the study was approved by the Bioethics and Welfare committee under the code P‐CBBA‐0121. Xenograft experiments were performed as described previously [30], injecting 1 × 10^6^ cells per tumour in the flank of 6/8 week‐old male athymic nude mice (Crl:NU(NCr)‐Foxn1^nu^). When tumours reached ~ 100 mm^3^, mice were randomly assigned to chow or doxycycline‐containing diets (no.: A153D70500; Ssniff Spezialdiäten GmbH), and tumour growth was monitored with an external calliper daily. Mice were fed *ad libitum* and housed under controlled environmental conditions, such as time‐controlled lighting on standard 12 : 12 light: dark cycles, controlled temperature at 22 ± 2 °C and 30–50% relative humidity. At the experimental endpoint, all mice were sacrificed by CO_2_ inhalation followed by cervical dislocation and tumours were excised, weighed and either snap‐frozen or fixed for further analyses.

### 2.8 Ubiquitination assay, co‐immunoprecipitation and immunoblotting

Ubiquitination assays were performed as described previously [28]. Briefly, HEK293T cells were co‐transfected with FLAG‐tagged ubiquitin together with the expression vector of the GFP‐tagged protein of interest. Cells were treated with MG132 (10 μm) for 2 h prior to lysis with lysis buffer [50 mm Tris–HCl pH 7.5, 150 mm NaCl, 1 mm EDTA, 0.5% Triton X‐100, 40 mm β‐Glycerolphosphate, 1 μg·mL^−1^ Leupeptin, 1 μg·mL^−1^ Aprotinin, 1 μg·mL^−1^ Pepstatin A and 7 mg·mL^−1^ N‐ethylmaleimide (NEM)]. Precleared lysates were incubated for 2.5 h at RT with prewashed GFP‐traps^®^ (Chromotek) and subsequently subjected to stringent washes in denaturing conditions (8 M urea, 1% SDS). Protein was eluted by boiling at 95 °C for 5 min (250 mm Tris–HCl pH 7.5, 40% glycerol, 4% SDS, 0.2% bromophenol blue, 5% β‐mercaptoethanol) and migrated on 4–15% Tris‐glycine gradient gels (Bio‐Rad).

Co‐immunoprecipitation assays were performed following the same procedure as for ubiquitination assays, but cells were lysed in the absence of NEM (50 mm Tris–HCl (pH 8), 120 mm NaCl, 1 mm EDTA, 1% IGEPAL CA‐630, 40 mm β‐Glycerophosphate, 1 μg·mL^−1^ Leupeptin, 1 μg·mL^−1^ Aprotinin and 1 μg·mL^−1^ Pepstatin A). Lysates were precleared by incubating with agarose beads (Chromotek) prior to overnight incubation with the GFP‐traps^®^, and mild washes were performed with detergent‐free nondenaturing lysis buffer. Protein complexes were eluted and migrated as described above.

For total cell extracts, cells were lysed with Laemmli buffer (50 mm Tris–HCl pH 6.8, 1.25% SDS, 15% glycerol) and total protein was quantified using the Lowry assay. Proteins were separated by sodium dodecyl sulphate‐polyacrylamide gel electrophoresis (SDS/PAGE) and transferred onto a PVDF membrane (Millipore). The following antibodies were used for immunoblotting: mouse anti‐β‐ACTIN (A5441; Sigma‐Aldrich), mouse anti‐CAIX (clone MN75; Bayer), mouse anti‐FLAG‐HRP (F3165; Sigma‐Aldrich), mouse anti‐GFP (11 814 460 001; Roche), mouse anti‐HA.11 (MMS‐101R; Covance), anti‐LC3 (2775 s; Cell Signaling Technology) and mouse anti‐MYC (9B11; Cell Signalling Technology). Home‐made rabbit anti‐HIF‐1α and anti‐PHD2 antibodies have been previously described [31]. Immunoreactive bands were visualized with ECL.

### 2.9 FLIM‐FRET (fluorescence lifetime imaging microscopy‐ förster/fluorescence resonance energy transfer)

Fluorescence lifetime images were acquired by scanning the sample with the LSM780 (Zeiss) scan head unidirectional and without averaging, recording a frame of 256 × 256 pixels with a pixel dwell time of 25.21 μs. Excitation of the green fluorescent donor fluorophore was controlled by the PDL 828 ‘Sepia II’ unit (PicoQuant) operating the 485‐nm pulsed diode laser (PicoQuant) with a repetition rate of 40 MHz. The objective used was a C‐Apochromat 40×/1.2 W Corr M27 (Zeiss). Fluorescence emission was collected through a 520/535 nm bandpass filter onto the Hybrid Detector PMA 40 (PicoQuant). The exact time between laser excitation and photon arrival was recorded using a time‐correlated single photon counting device (TCSPC), specifically the TimeHarp260 (PicoQuant). The data were then plotted in a histogram to create a fluorescence decay curve. An instrument response function (IRF) using erythrosine B was daily recorded under the same measurement conditions as described previously [32]. SymPhoTime 64 software (PicoQuant) controlled all PicoQuant hardware devices and was used for analysis. All photons within the region of interest were included in the lifetime fitting analysis. The TCSPC‐curve was reconvoluted with the IRF and fitted to a two‐component decay curve to extract average lifetimes τ_Av Int_.

### 2.10 Bioinformatics analysis and statistics

Prostate cancer data retrieved from Taylor *et al*. [33] have been subjected to background correction, log2 transformation and quartile normalization. For correlation analysis, a Spearman's correlation test was applied. Spearman's coefficient (ρ) indicates the existing linear correlation (dependence) between two variables, X and Y, giving a value between +1 and −1 (both included), where 1 is total positive correlation, 0 is no correlation, and −1 is total negative correlation. The *P*‐value indicates the significance of this *ρ* coefficient. Individual gene expression patterns in different pathophysiological statuses were performed using the web‐based interface Cancertool [34].

*n* values represent the number of independent experiments performed, the number of individual mice or patient specimens. Data represent mean ± SEM/SD or 95% CI of pooled experiments except for the western blots corresponding to a representative replicate of at least three independent experiments. We assumed a normal distribution in each independent *in cellulo* experiment. A one‐sample *t*‐test with the corresponding hypothetical value 1 was used for one‐component comparisons with control, a student's *t*‐test was utilized for two‐component comparisons and, an ANOVA test was used for comparisons involving multi‐components. In the case of *in vivo* experiments and patient data, a nonparametric Mann–Whitney *U* or Kruskal–Wallis test was used. The confidence level used for all the statistical analyses was 0.95 (alpha value = 0.05). A two‐tailed statistical analysis was applied for data mining analysis. GraphPad Prism 8 software was used for statistical calculations: *P < 0.05; **P < 0.005; ****P < 0.0001; ^#^ *P* < 0.05; ^##^ *P* ≤ 0.005; *P* > 0.05 ns (nonsignificant).

## 3 Results

To identify hypoxia‐related DUBs involved in PCa progression, we first sought to identify bona fide hypoxia/HIF‐target genes associated with disease aggressiveness in publicly available transcriptomics databases of PCa patients [33]. We found that CA9, an established HIF target [35], was upregulated in primary tumours with high Gleason Score (GS), a method used in the clinics to stratify patients and predict their prognosis [36], and in metastatic specimens when compared to noncancerous prostate (Fig. S1A). In turn, we carried out an unbiased expression correlation study of CA9 and the catalogue of DUBs (Fig. 1A and Table S1).

**Fig. 1:**
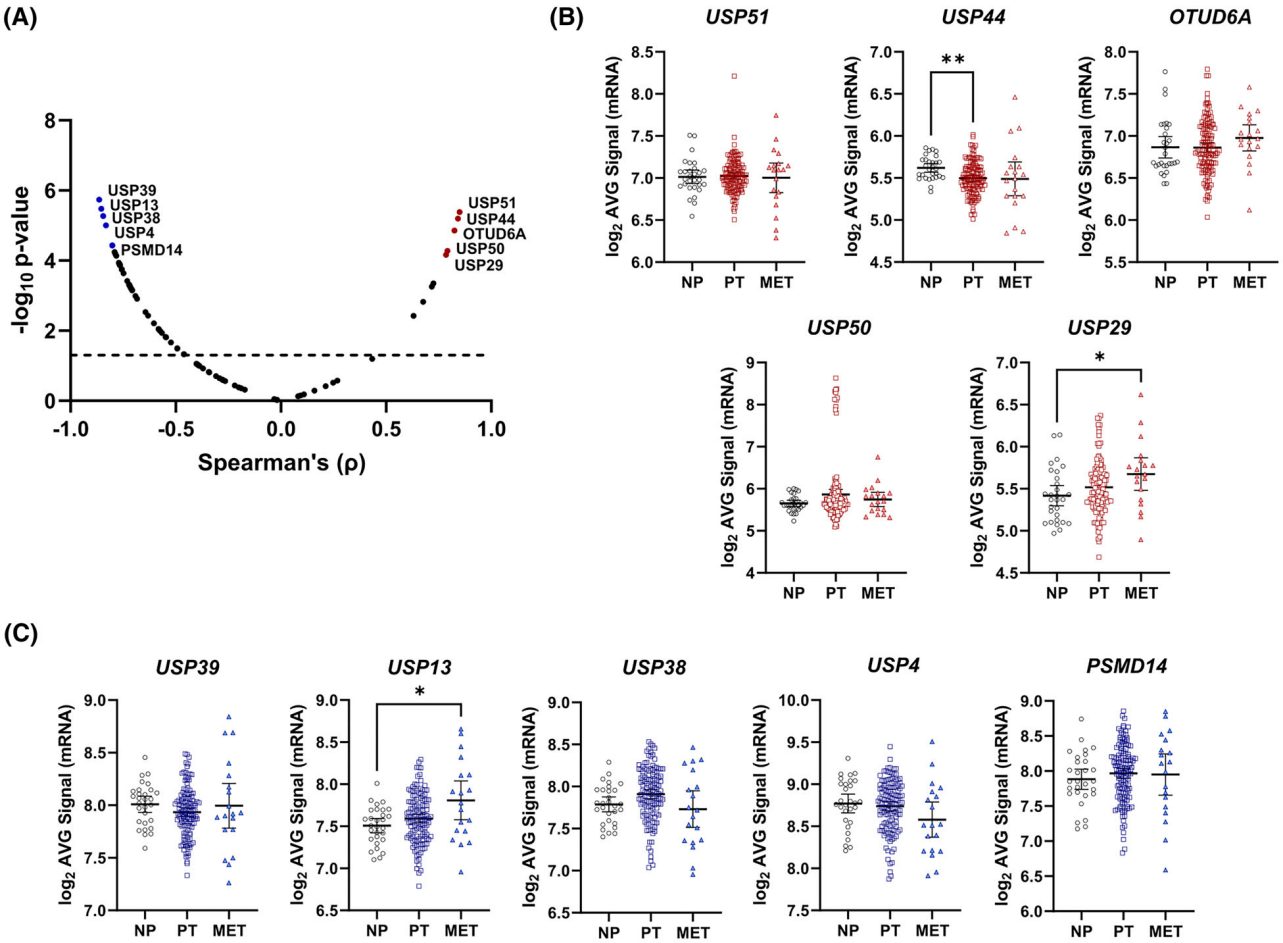
*USP29* correlates with *CA9* and tumour aggressiveness in prostate cancer (PCa) patients. (A) Scatter plot displaying the correlation between *CA9* (carbonic anhydrase) and the different *DUB* (deubiquitinating enzymes) mRNA levels in Taylor *et al*. dataset of metastatic PCa samples [25]. Spearman's correlation test (*ρ*) was used for statistical analysis and −Log_10_‐*P*‐values were indicated at the *y*‐axis. (B, C) Gene expression analysis of the TOP‐5 *DUBs* that positively (B) and negatively (C) correlated with *CA9* in the aforementioned dataset of PCa samples. *DUB* mRNA levels in prostate samples were compared based on the tissue origin of the patient [nontumoural tissue (NP): *n* = 29, primary tumours (PT): *n* = 131; metastatic tumours (MET): *n* = 19]. The y‐axis represents the Log_2_‐normalized gene expression. *P*‐value derives from the Kruskal–Wallis test between the indicated groups (*P*, *P*‐value: *P < 0.05, **P < 0.005). Error bars represent 95% CI (confidence interval).

The detailed analysis of the TOP‐10 candidates (TOP‐5 DUBs showing the highest positive and negative Spearman's ρ correlation with CA9, respectively) revealed that USP29 was the only DUB fulfilling the selection criteria and mirroring the expression profile of CA9 (Figs 1B,C and Fig. S1B,C). The expression levels of USP29 mRNA were progressively increased from nontumoural prostate tissue over the primary tumour to metastasis and exhibited a significant association with the GS.

To address the role of USP29 in PCa biology, we modulated USP29 expression levels in a metastatic PCa cell line (PC3). First, we silenced USP29 in a doxycycline‐inducible manner using two different short hairpin RNAs (Fig. S2A). The silencing of USP29 did not have an impact on the bi‐dimensional growth or the clonogenic capacity of the cells (Fig. 2A and Fig. S2B). In line with this, the inducible silencing of USP29 did not alter the growth of subcutaneous tumours transplanted in the flanks of immunocompromised mice (Fig. 2B and Fig. S2C). Of note, we excluded the possibility that doxycycline treatment could influence the results (Fig. 2A,B and Fig. S2A). Next, we overexpressed USP29 in PC3 cells (Fig. S2D). We did not observe a contribution of USP29 to PCa cells on bi‐dimensional growth, colony formation or cell cycle progression (Fig. 2C and Fig. S2E & F). However, expression of USP29 did promote faster wound closure in wound‐healing migration assays (Fig. 2D and Fig. S2G), suggesting a role for USP29 in PCa cell migration, rather than cell proliferation. In line with these results, expression of USP29 increases the expression of *ZEB2 (*zinc finger E‐box‐binding homeobox 2) and *SNAI2*, two markers of the epithelial‐to‐mesenchymal transition (EMT) promoting cell migration capacity (Fig. S2H). Moreover, USP29 expression attenuated the wound closure‐promoting effect of the pan‐hydroxylase inhibitor DMOG (Fig. 2E).

**Fig. 2:**
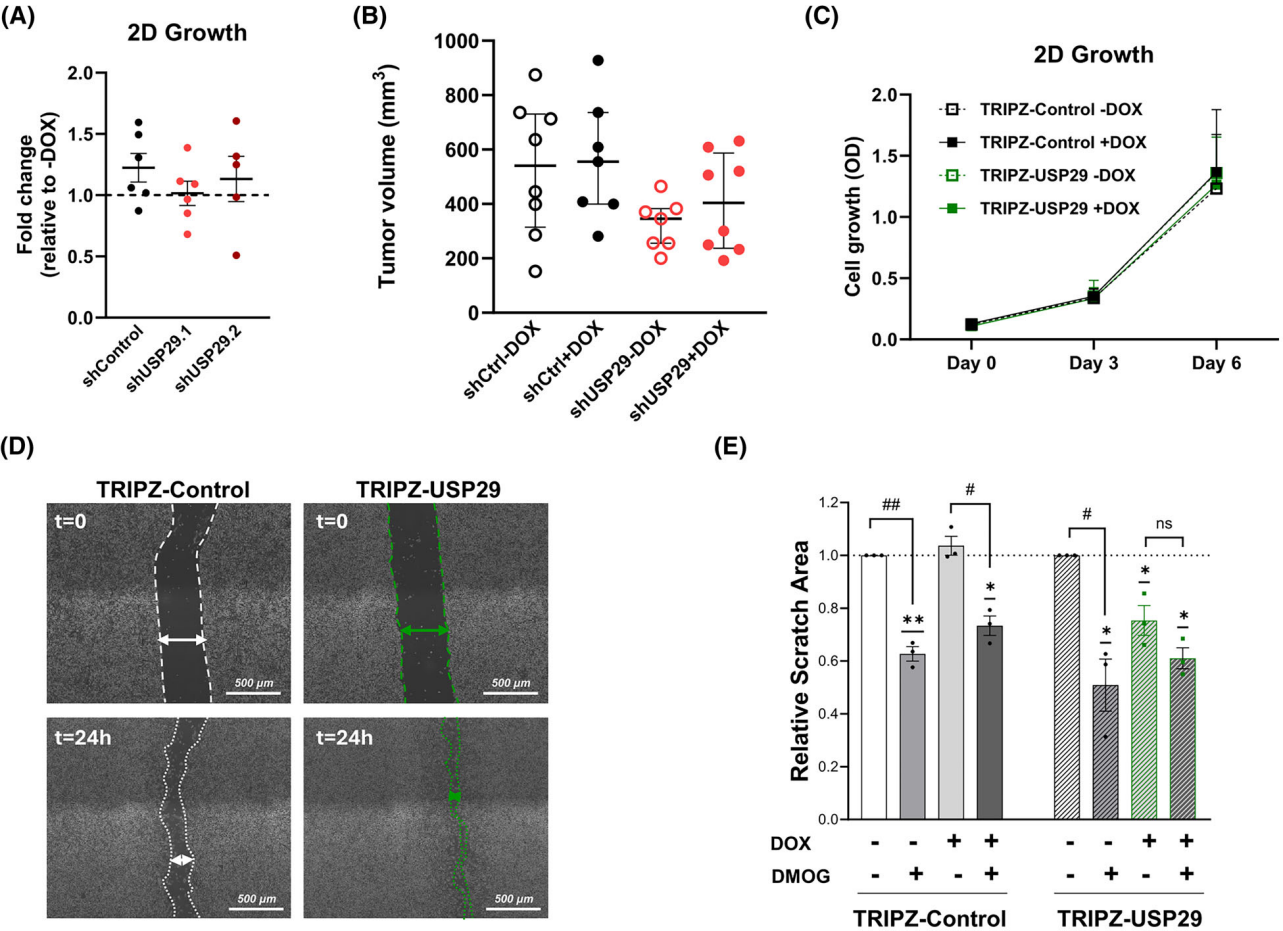
Impact of USP29 expression in prostate cancer (PCa) cell lines *in cellulo* and *in vivo*. (A) Proliferation of PC3 cells silenced with control or two different shRNAs targeting USP29 (ubiquitin specific protease 29; shUSP29.1 and shUSP29.2) in an inducible manner (DOX, doxycycline, 0.5 μg·mL^−1^). Data are represented as cell number at Day 6 relative to ‐DOX cells. Statistical analysis: One‐sample *t*‐test (hypothetical value = 1; *P*‐value > 0.05). Error bars represent SEM. shControl (*n* = 6), shUSP29.1 (*n* = 6), and shUSP29.2 (*n* = 5) independent experiments. (B) Quantification of tumour xenograft size at experimental endpoint following injection of 1 × 10^6^ PC3 cells silenced with shControl or shUSP29.1 in an inducible manner. Kruskal–Wallis test was used for statistical analysis. Error bars represent 95% CI (confidence interval; *n* = 7–8; *P*‐value > 0.05). (C) Growth curve (relative to Day 0) of PC3 cells in the absence or the presence of 0.5 μg·mL^−1^ DOX to induce the ectopic expression of USP29 (*n* = 3 independent experiments). Two‐way ANOVA test was used for statistical analysis. Error bars represent SD (*P*‐value > 0.05). (D) Representative images corresponding to a wound‐healing assay (*n* = 3 independent experiments) of control and USP29 overexpressing PC3 cells in the presence of 0.5 μg·mL^−1^ DOX at two different time points (*t* = 0 and *t* = 24 h). Forty‐eight hours after DOX addition were defined as the initial timepoint for the assay. Dashed lines indicate the wound edges (white in control cells and green in USP29 overexpressing cells). Arrows denote the distance between the two wound margins. White scale bars = 500 μm. (E) Quantification of the impact of dimethyloxalylglycine (DMOG) on PC3 cell migration. Data are represented as the relative scratch area at 8 h of control and USP29 overexpressing treated or not with DMOG (1 mm). One‐sample *t*‐test (hypothetical value = 1; *P*‐value: *P < 0.05, **P < 0.005) and paired *t*‐test (*P*‐value: ns, *P* > 0.05, ^#^ *P* < 0.05, ^##^ *P* < 0.005) were used for statistical analysis. Error bars represent SEM (*n* = 3 independent experiments).

Our screening was conceived to identify DUBs that regulate hypoxia‐dependent transcription. Therefore, we sought a deeper understanding of the mechanism underpinning USP29‐mediated HIF signalling. *USP29* silenced cells exhibited reduced accumulation of HIF‐1α protein upon hypoxia, together with lower levels of its downstream targets CAIX and PHD2 (Fig. 3A). Conversely, ectopic expression of USP29 promoted the accumulation of endogenous HIF‐1α, CAIX and PHD2 in normoxia (Fig. 3B). In parallel, DMOG was used as a positive control to ensure proper stabilization of HIF pathway components. Surprisingly, silencing of endogenous *USP29* decreased the protein levels of both HIF‐1α WT and HIF‐1α DM^(PP/AA)^, a HIF‐1α mutant whose two canonical oxygen‐sensitive proline residues have been replaced by alanine (P402/564A) (Fig. 3C and Fig. S3A). Consistently, the ectopic expression of USP29 also accumulated HIF‐1α DM^(PP/AA)^ (Fig. 3D). In contrast, silencing of the bona fide negative regulators, *PHD2/EGLN1* and *pVHL* [12, 31] only affected HIF‐1α WT but not HIF‐1α DM^(PP/AA)^ (Fig. 3C). Taken together, these results indicate for the first time that USP29 acts on HIF‐1α through a noncanonical mechanism.

**Fig. 3:**
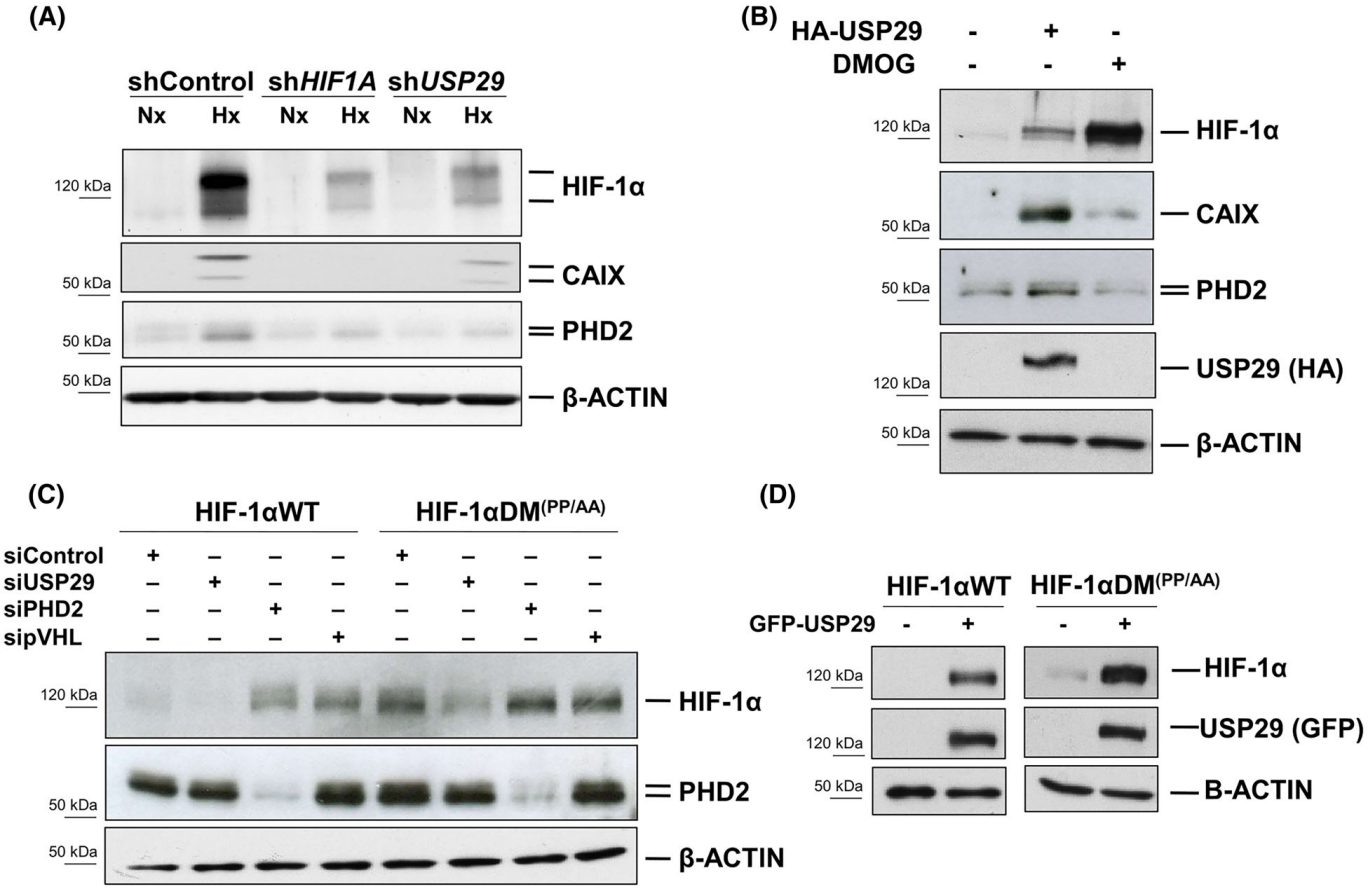
USP29 regulates HIF‐1α in a non‐canonical way. (A) Representative western blot analysis of HeLa cells silenced with shRNAs (shControl, sh*HIF1A* or sh*USP29*) and incubated for 4 h in normoxia (Nx; 21% O_2_) or hypoxia (Hx; 1% O_2_). Whole‐cell extracts (WCE) were subjected to sodium dodecyl sulphate‐polyacrylamide gel electrophoresis (SDS/PAGE) followed by immunoblotting with the indicated antibodies (*n* = 3 independent experiments). (B) Representative western blot analysis of HEK293T cells transfected with empty vector or HA‐USP29 and left untreated or treated with the hypoxia mimetic DMOG (1 mm) for 4 h prior to lysis. WCE were subjected to SDS/PAGE and immunoblotting was performed using the indicated antibodies (*n* = 3 independent experiments). (C) Representative western blot analysis of HEK293T cells silenced with control or siRNAs targeting endogenous *USP29*, *EGLN1/PHD2* or *pVHL*, and transfected with Myc‐HIF‐1α or Myc‐HIF‐1α DM^(PP/AA)^. WCE were subjected to SDS/PAGE followed by immunoblotting with the indicated antibodies (*n* = 3 independent experiments). (D) Representative western blot analysis of HEK293T cells co‐transfected with Myc‐HIF‐1α WT or Myc‐HIF‐1α DM^(PP/AA)^ and empty vector or GFP‐USP29. WCE were subjected to SDS/PAGE followed by immunoblotting with the indicated antibodies (*n* = 3 independent experiments).

To gain further insight into how USP29 elicits the accumulation of HIF‐1α, we generated a catalytically inactive USP29 mutant by replacing its active site cysteine residue C294 with a serine (USP29^C/S^) [37]. This mutation completely abrogated the ability of USP29 to promote the accumulation of HIF‐1α DM^(PP/AA)^ (Fig. 4A), pointing towards a crucial role of the ubiquitin specific peptidase activity of USP29 in the noncanonical HIF‐1α regulation. Next, we explored the requirements of the two main protein degradation processes, namely the proteasome and the lysosome. We treated cells with the proteasome inhibitor MG132 or the lysosome disruptor chloroquine for 4 h and monitored the consequences on HIF‐1α DM^(PP/AA)^ protein levels alone or in combination with USP29 ectopic expression. Interestingly, only the proteasome inhibition stabilized HIF‐1α DM^(PP/AA)^ (Fig. 4B and Fig. S4A). Moreover, MG132 did not exacerbate the effect of USP29 overexpression, suggesting that they both acted on the same pathway. Cycloheximide experiments showed that USP29 increased the half‐life of HIF‐1α DM^(PP/AA)^ (Fig. S4B). More importantly, USP29 stabilized endogenous HIF‐1α upon reoxygenation (Fig. 4C). Expression of USP29 extended the presence of a fraction of HIF‐1α for more than 60 min, whereas in control cells, the protein was no longer detectable 30 min after reoxygenation.

**Fig. 4:**
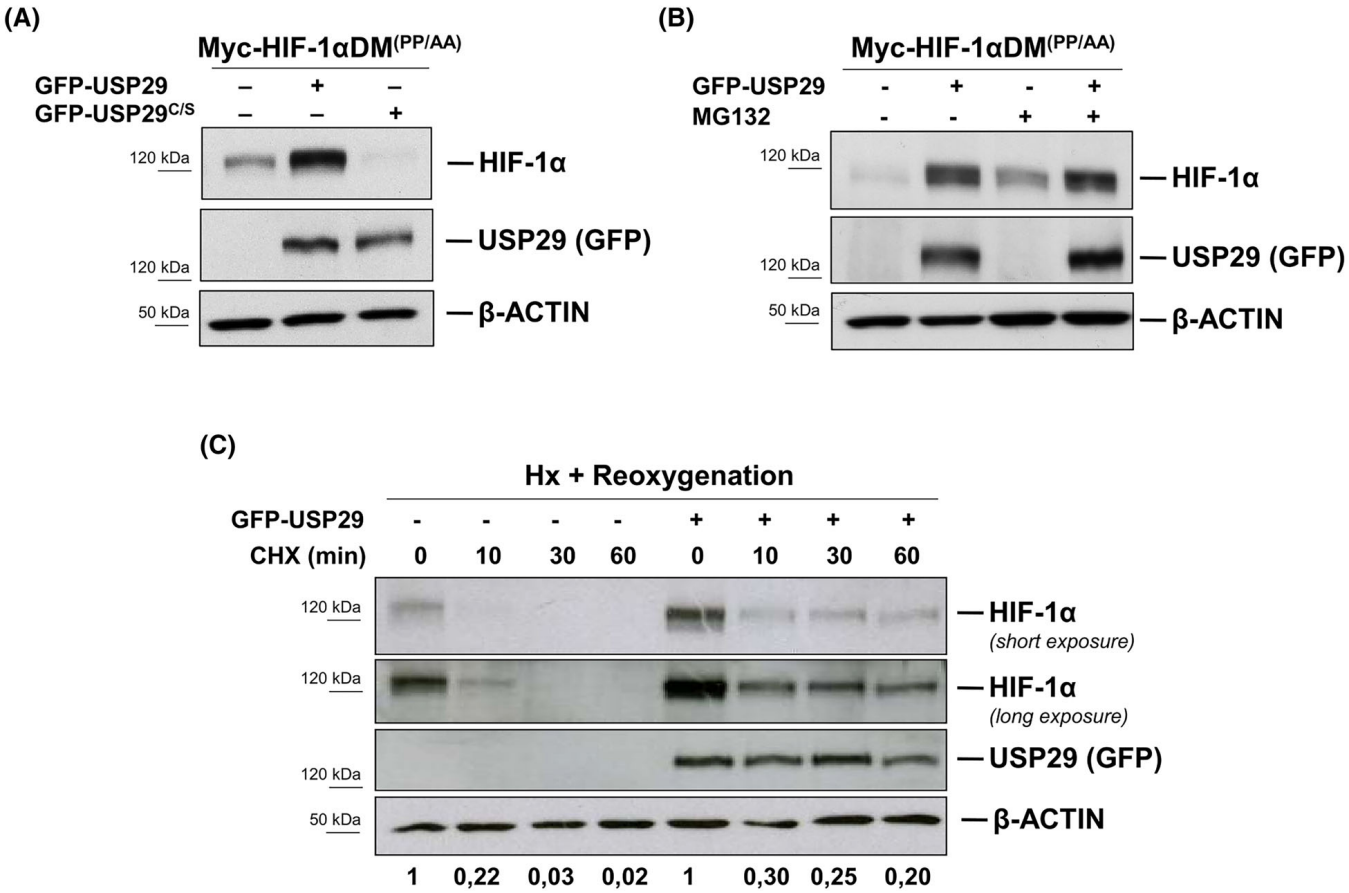
Catalytically active USP29 stabilizes HIF‐1α by protecting it from proteasome‐mediated degradation. (A) Representative western blot analysis of HEK293T cells co‐transfected with Myc‐HIF‐1α DM^(PP/AA)^ and empty vector, GFP‐USP29 or the catalytically inactive mutant, GFP‐USP29^C/S^. Whole‐cell extracts (WCE) were subjected to immunoblotting with the indicated antibodies (*n* = 3 independent experiments). (B) Representative western blot analysis of HEK293T cells co‐transfected with Myc‐HIF‐1α DM^(PP/AA)^ and empty vector or GFP‐USP29 and left untreated or treated with the proteasome inhibitor MG132 (10 μm) for 4 h. WCE were subjected to SDS/PAGE followed by immunoblotting with the indicated antibodies (*n* = 3 independent experiments). (C) Representative western blot analysis of HEK293T cells transfected with empty vector or GFP‐USP29, incubated in hypoxia (1% O_2_) for 4 h, and afterwards treated with cycloheximide (CHX, 20 μg·mL) to inhibit protein synthesis and reoxygenated. WCE were prepared at the indicated time points and analysed by immunoblotting with the indicated antibodies (*n* = 3 independent experiments).

The effect of USP29 on HIF‐1α DM^(PP/AA)^ was further extended to a variety of cell lines of different origins, including PC3 and LnCaP (PCa), A2780 (ovarian cancer), SH‐SY5Y and SK‐N‐AS (neuroblastoma), and MDA‐MB‐231 (breast cancer). The overexpression of USP29 led to an increase in HIF‐1α DM^(PP/AA)^ levels in all tested cell lines (Fig. 5A), indicating that this regulation is likely a general phenomenon. Consistent with our *in cellulo* findings and prior studies, USP29 amplification was observed in multiple tumour types according to data from the TCGA consortium, accessed through cBioPortal (Fig. 5B) [38, 39]. Interestingly, not only HIF‐1α but also the wild type and the oxygen‐insensitive DM^(PP/AA)^ forms of HIF‐2α/EPAS‐1 accumulated upon overexpression of USP29 (Fig. 5C).

**Fig. 5:**
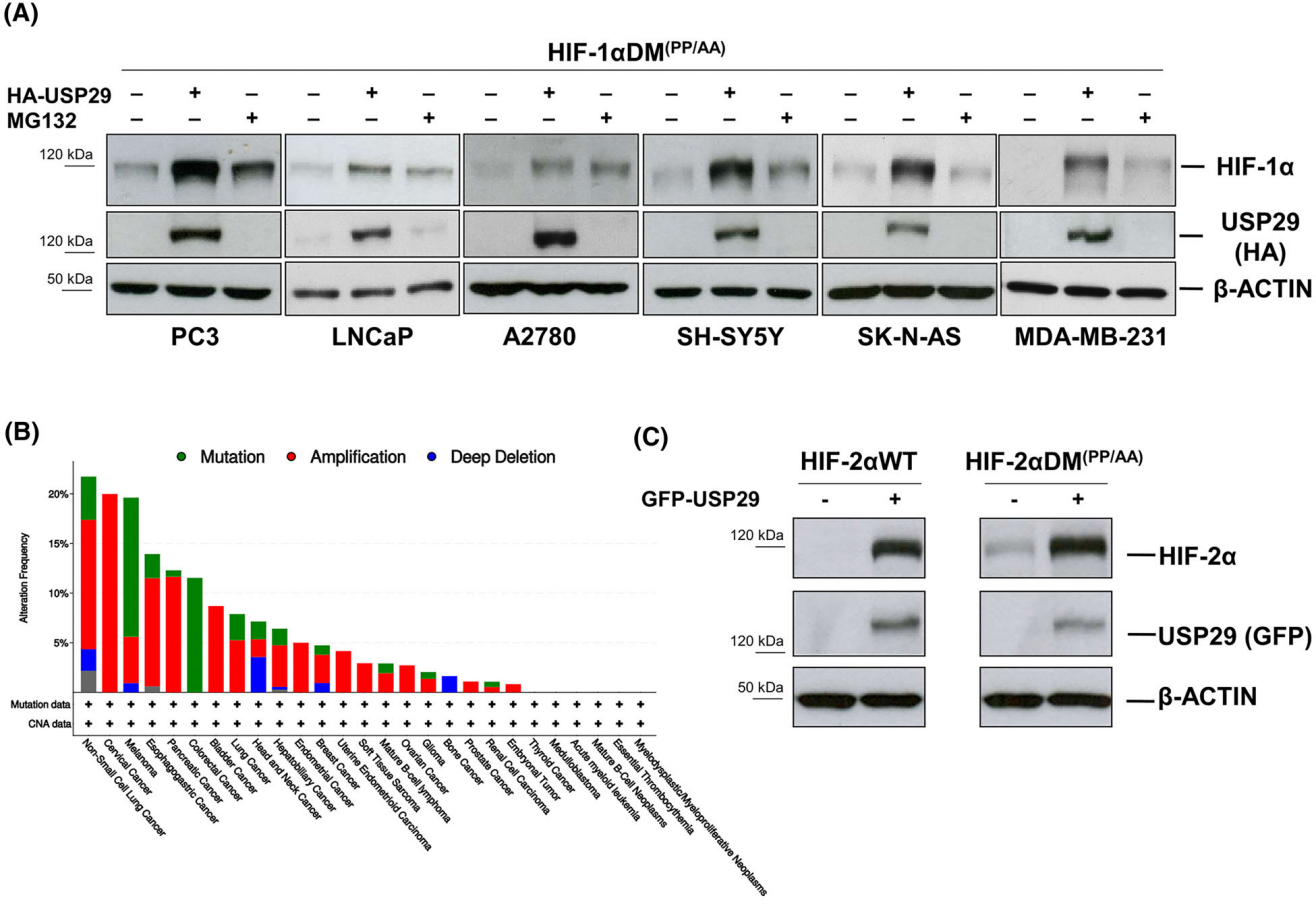
Wide impact of USP29 on HIF‐α. (A) Representative western blot analysis of whole‐cell extracts (WCE) arising from cancer cell lines of different origins co‐transfected with Myc‐HIF‐1α DM^(PP/AA)^ and empty vector or HA‐USP29 and left untreated or treated with the proteasome inhibitor MG132 (Z‐L‐Leu‐D‐Leu‐L‐Leu‐al, 10 μm) for 4 h (*n* = 3 independent experiments). (B) Analysis of USP29 genetic alterations (frequency and type of alterations) in diverse types of cancer using cBioPortal data. (C) Representative western blot analysis of HEK293T cells co‐transfected with Myc‐HIF‐2α or Myc‐HIF‐2α DM^(PP/AA)^ and empty vector or GFP‐USP29. WCE were analysed by immunoblotting with the indicated antibodies (*n* = 3 independent experiments).

As the catalytical activity of USPs is responsible for removal of (poly)ubiquitin chains from their target proteins, we next tested whether USP29 was able to function as a DUB for HIF‐α DM^(PP/AA)^. We first analysed USP29 and HIF‐α DM^(PP/AA)^ interaction by co‐immunoprecipitation. We immunoprecipitated GFP‐tagged HIF‐1α DM^(PP/AA)^ or GFP alone from HEK293T cells, and we found HA‐USP29 interacting with GFP‐tagged HIF‐1α DM^(PP/AA)^, but not with GFP alone (Fig. 6A). To rule out a nondirect interaction, we confirmed these data by using fluorescence lifetime‐based FRET measurements (Fig. 6B). The fluorescence lifetime of the FRET donors, HIF‐1α DM^(PP/AA)^‐Clover, was significantly decreased from 2.85 ± 0.02 ns to 2.70 ± 0.09 ns in the presence of the FRET acceptor mCherry‐USP29. As FRET only occurs when both fluorophores are in close proximity (around 6 nm), these data demonstrate that USP29 interacts with HIF‐1α DM^(PP/AA)^. Comparable results were obtained when we analysed the interaction between USP29 and HIF‐2α DM^(PP/AA)^ (Fig. S5A). The lifetime of HIF‐2α DM^(PP/AA)^‐GFP was significantly reduced from 2.39 ± 0.01 ns to 2.28 ± 0.06 ns in the presence of mCherry‐USP29. Next, we co‐transfected GFP‐tagged HIF‐1α DM^(PP/AA)^ together with FLAG‐ubiquitin either in the absence or the presence of HA‐USP29 or HA‐USP29^C/S^. After the enrichment of the ubiquitinated proteome by MG132 treatment, GFP‐HIF‐1α DM^(PP/AA)^ was pulled down under highly denaturing conditions and anti‐FLAG‐antibody was used to detect ubiquitinated GFP‐HIF‐1α DM^(PP/AA)^. We found that wild type USP29, but not the catalytically inactive USP29^C/S^, markedly decreased the basal ubiquitination of HIF‐1α DM^(PP/AA)^ and increased the nonmodified population of HIF‐1α DM^(PP/AA)^ (Fig. 6C). Consistently, *USP29* silencing increased poly‐ubiquitination of HIF‐1α DM^(PP/AA)^ (Fig. S5B), pointing towards an existing basal deubiquitinating activity of endogenous USP29. Expression of a siRNA‐resistant USP29 restored the basal HIF‐1α DM^(PP/AA)^ ubiquitination pattern (Fig. S5B right lane). Furthermore, USP29 also exerted deubiquitination activity towards HIF‐2α DM^(PP/AA)^ (Fig. S5C). Taken together, our results indicate that endogenous and ectopic USP29 is an efficient DUB for HIF‐α DM^(PP/AA)^, thereby increasing HIF‐α stabilization through a PHDs/pVHL‐independent mechanism.

**Fig. 6:**
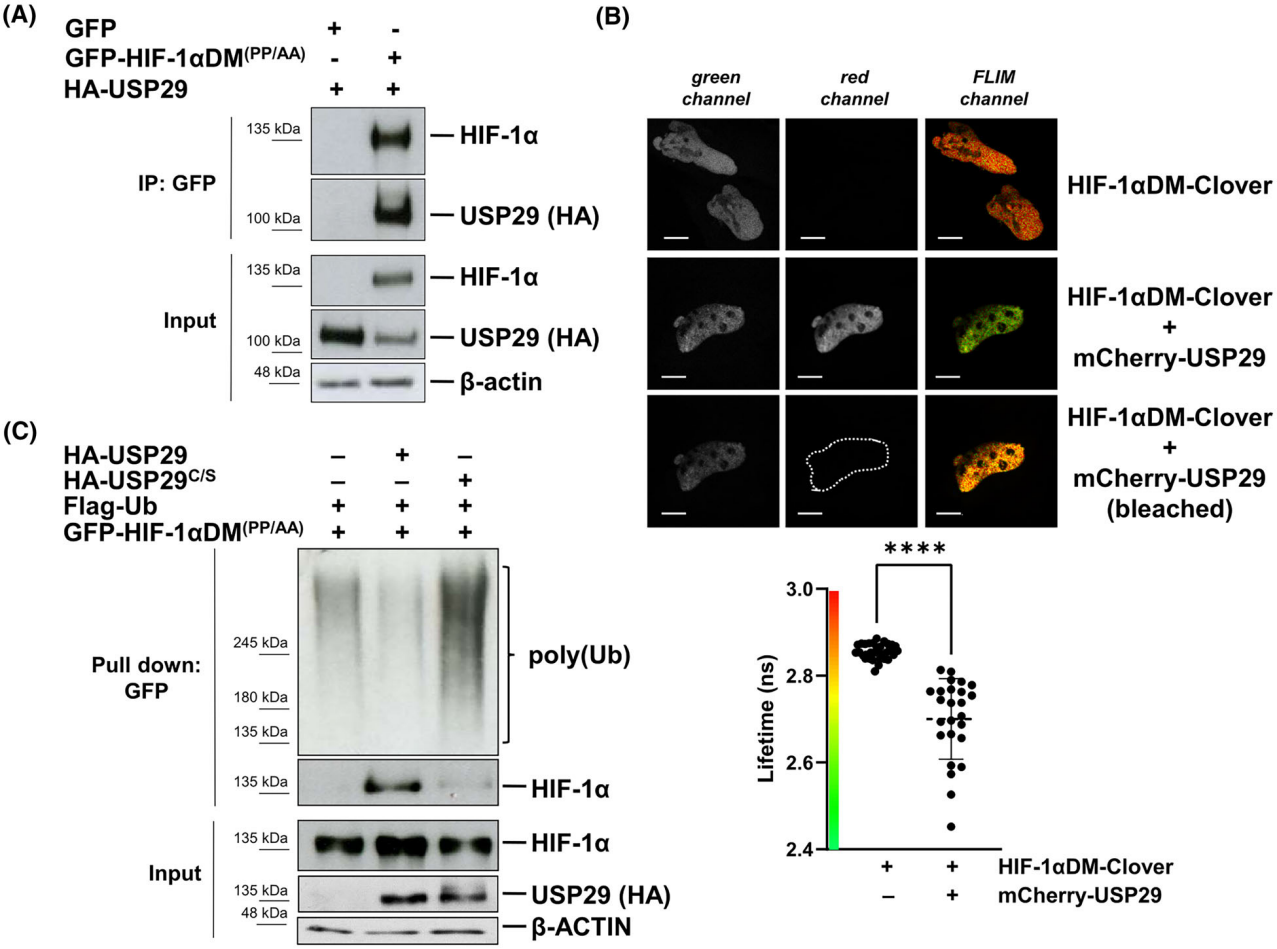
USP29 interacts with and deubiquitinates HIF‐α DM^(PP/AA)^. (A) Representative western blot analysis of HEK293T cells co‐transfected with HA‐USP29 and either GFP alone or GFP‐HIF‐1α DM^(PP/AA)^. Cells were lysed in native conditions and GFP‐tagged proteins were immunoprecipitated with GFP‐traps^®^. Immuno‐complexes were analysed for the presence of HA‐USP29 by immunoblotting (*n* = 3 independent experiments). (B) Fluorescence images acquired from HeLa cells transfected with the FRET (föster/fluorescence resonance energy transfer) donor (HIF‐1α DM^(PP/AA)^‐Clover green channel, left panel) alone or together with the FRET acceptor (mCherry‐USP29; red channel, central panel). The lifetime of the donor was measured, and pseudo‐colour coded fluorescence lifetime images (FLIM channel, right panel) were generated. Data, from three independent experiments, are represented as average lifetime of the donor in the absence (*n* = 33) and the presence (*n* = 25) of the FRET acceptor. Unpaired *t*‐test was used for statistical analysis (*P*‐value: ****P < 0.0001). Error bars represent SD. Scale bars are 10 μm long. (C) Representative western blot analysis of HEK293T cells co‐transfected with GFP‐HIF‐1α DM^(PP/AA)^, FLAG‐ubiquitin and either HA‐USP29 or HA‐USP29^C/S^. Cells were treated with the proteasome inhibitor MG132 (10 μm) for 2 h and lysed in the presence of the DUB inhibitor N‐ethylmaleimide (NEM, 7 mg·mL^−1^). GFP‐HIF‐1α DM^(PP/AA)^ was pulled down with GFP‐traps^®^ and subjected to stringent washes (8 M urea, 1% SDS). Ubiquitinated and nonubiquitinated GFP‐HIF‐1α DM^(PP/AA)^ protein in the eluate was analysed by immunoblotting with anti‐FLAG and anti‐GFP antibodies, respectively (*n* = 3 independent experiments).

To identify the potential lysine residues targeted by USP29's deubiquitinating activity, we assessed truncated forms of HIF‐1α DM^(PP/AA)^ for their susceptibility to USP29. The N‐terminal part, HIF‐1α DM^1‐657^, was not affected by the presence of USP29, while the C‐terminal end (HIF‐1α^630‐826^) accumulated in the presence of USP29, similar to the full‐length protein (Fig. 7A). The USP29^C/S^ mutant that lacked catalytic activity was not able to accumulate HIF‐1α^630‐826^ (Fig. S6A). Correspondingly, USP29 acted also on the C terminus of HIF‐2α (Fig. S6B). We used truncations of the C terminus to further delineate the USP29 target site within HIF‐1α. HIF‐1α^630‐713^ and HIF‐1α^630‐750^ were resistant to USP29‐mediated accumulation (Fig. S6C) and pointed out the importance of the very C‐terminal tail of HIF‐1α for this regulation. This tail contains two evolutionarily conserved lysines (K752 and K755), which are also shared by HIF‐2α and a neighbouring lysine (K758) (Fig. S6D). In fact, cycloheximide experiments confirmed that the mutation of all three evolutionarily conserved lysines to arginines (HIF‐1α DM^KKK/RRR^) conferred a higher stability to the protein (Fig. 7B). Importantly, the basal ubiquitination of HIF‐1α DM^KKK/RRR^ was significantly reduced compared with HIF‐1α DM (Fig. 7C).

**Fig. 7:**
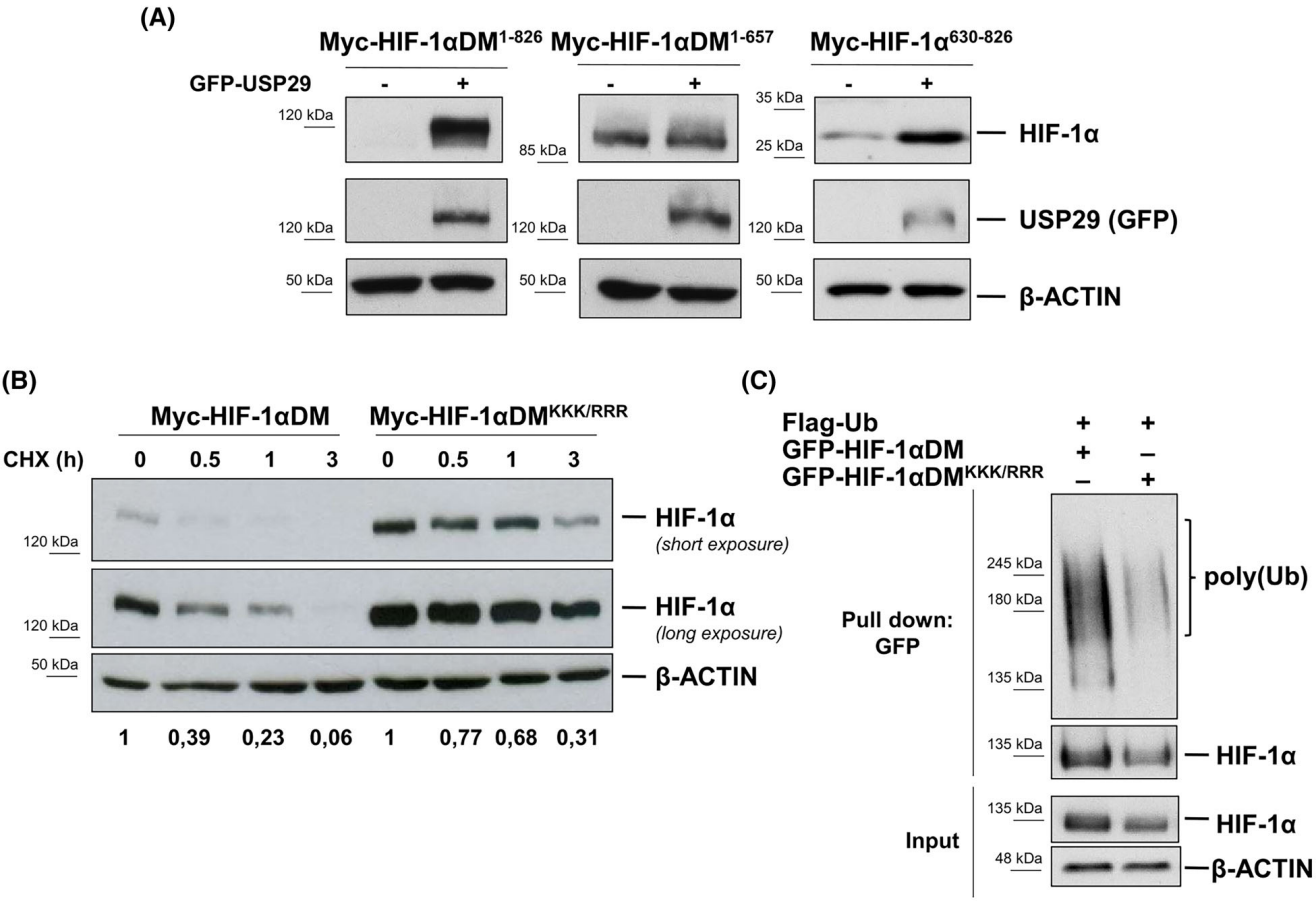
USP29 targets the C‐terminal part of HIF‐α. (A) Representative western blot analysis of HEK293T cells co‐transfected with Myc‐HIF‐1α DM^1‐826^, Myc‐HIF‐1α DM^1‐657^ or Myc‐HIF‐1α DM^630‐826^ and either empty vector or GFP‐USP29. Whole‐cell extracts (WCE) were subjected to immunoblotting with the indicated antibodies (*n* = 3 independent experiments). (B) Representative western blot analysis of HEK293T cells transfected with Myc‐HIF‐1α DM or Myc‐HIF‐1α DM^KKK/RRR^ and treated with cycloheximide (CHX, 20 μg·mL^−1^) to inhibit protein synthesis. WCE were collected at the indicated times after CHX treatment and protein levels of the Myc‐tagged HIF‐1α proteins analysed (*n* = 3 independent experiments). (C) Representative western blot analysis of HEK293T cells co‐transfected with GFP‐HIF‐1α DM or Myc‐HIF‐1α DM^KKK/RRR^ and FLAG‐ubiquitin. Cells were treated with the proteasome inhibitor MG132 (10 μm) for 2 h and lysed in the presence of the DUB inhibitor NEM (7 mg·mL^−1^). GFP‐HIF‐1α was pulled down with GFP‐traps^®^ and subjected to stringent washes (8 M urea, 1% SDS). Ubiquitinated and nonubiquitinated GFP‐HIF‐1α protein in the eluate was analysed by immunoblotting with anti‐FLAG and anti‐GFP antibodies, respectively (*n* = 3 independent experiments).

## 4 Discussion

Our results identify USP29 as the only DUB closely mirroring CA9 expression and associated with PCa aggressiveness in patient samples. This correlation, along with the progressive increase of USP29 expression from benign tissue to metastases, is consistent with a potential oncogenic role for USP29 in PCa, although causality remains to be firmly established.

Functionally, although USP29 did not impact proliferation or tumour growth, it reproducibly promoted migration and EMT marker expression consistently with the high USP29 expression found in metastatic prostatic tumours. The pro‐tumoural role of USP29 has recently been explored in cancer [40, 41, 42, 43, 44, 45, 46, 47]. Our results align with the reported association of high expression levels of USP29 with malignancy in colorectal, gastric and triple‐negative breast cancer [40, 41, 42]. Furthermore, USP29 mediates chemotherapy‐induced resistance in nonsmall lung cancer (NSCLC), hepatocellular carcinoma (HCC) and triple‐negative breast cancer (TNBC) [42, 43, 44]. Conversely, our results apparently contradict previous studies reported in colorectal cancer, which showed an inhibitory impact of USP29 silencing on cancer cell proliferation and xenograft tumour growth [40]. Overall, the results reveal a reproducible yet modest influence of USP29 on PCa cell behaviour, compatible with a context‐dependent modulatory function. This modest phenotypic output aligns with the extensive redundancy and compensatory mechanisms characteristic of PCa signalling networks.

Mechanistically, our findings point to USP29 as an upstream activator of HIF‐1α. To date, USP29 has been reported to exhibit DUB activity towards p53, CLASPIN, SNAIL, CDC25, NRF2, TWIST, KIAA1429, MYC and HIF‐1α [29, 37, 41, 42, 43, 44, 45, 46, 47, 48]. Indeed, USP29‐mediated glycolysis upregulation has been associated with Sorafenib resistance of HCC through HIF‐1α stabilization, though the underlying mechanism was not yet fully understood [44]. Our data demonstrate that USP29 stabilizes HIF‐α through a noncanonical, proteasome‐dependent, PHD/pVHL‐independent mechanism. This distinguishes USP29 from classical regulators of HIF stability, as USP29 acts directly on the C‐terminal lysines of HIF‐α, a region previously unassociated with HIF stabilization [21, 22]. Our findings suggest that USP29‐mediated modulation of HIF‐α stability likely operates in parallel with canonical regulation and may adjust the pool of hydroxylated HIF‐α depending on the surrounding conditions. Of note, USP29 stabilizes not only HIF1α but also HIF2α, which remains considerably less characterized. Indeed, Cezanne/OTUD7B, USP5, USP8 and USP33 have been described to regulate HIF‐2α [24]. However, excluding Cezanne, which indirectly regulates EPAS1 transcript, there is no conclusive mechanistic information about such regulation [49].

The identification of USP29 as a noncanonical regulator, together with its upregulation in aggressive PCa, suggests that elevated USP29 activity may contribute to the sustained HIF‐α accumulation observed in prostate tumours even under oxygen‐replete conditions. In this scenario, USP29 could help maintain HIF signalling and favour migratory behaviours linked to tumour progression. Although our data do not demonstrate causality *in vivo*, the reproducible effects and mechanistic insights strongly suggest that USP29 acts as a fine‐tuning context‐dependent regulator operating alongside canonical pathways. Future studies, including *in vivo* assessment of metastatic potential and deeper analysis of C‐terminal HIF‐α ubiquitination and its interplay with FIH‐mediated hydroxylation, should elucidate the extent to which USP29 shapes HIF signalling and PCa behaviours.

## 5 Conclusions

Our study identifies Ubiquitin‐Specific Protease 29 (USP29) as the sole DUB whose expression mirrors that of CA9, a surrogate marker of intratumoural hypoxia and HIF pathway activation associated with PCa aggressiveness. Mechanistically, we demonstrate that USP29 enhances hypoxia signalling by stabilizing HIF‐1α and HIF‐2α through their C‐terminal region via a noncanonical, PHD/pVHL‐independent yet proteasome‐dependent mechanism, thereby positioning USP29 as a previously unrecognized regulator of hypoxic responses in PCa.

## Conflict of interest

The authors declare that they have no conflict of interest.

## Author contributions

ASS designed and performed most of the experiments to understand the molecular mechanisms behind USP29‐mediated HIF‐signalling regulation and contributed to the analysis of the data and the writing of the manuscript. LMC and LMP manipulated USP29 levels in PC3 cells and executed *in cellulo* and *in vivo* experiments with the assistance of AE and AZL, respectively. AVB, BCC, EPA, OC and SP provided support with the generation of molecular tools and cellular assays. MFE provided support with the preparation of the manuscript. SGL and IM provided support for the bioinformatic analysis. UM, AC and VS provided technical support and contributed to the critical discussion of the results and manuscript revision. EB designed and supervised the project, analysed data and wrote the manuscript.

## Supporting information


**Fig. S1.** *USP29* correlates with hypoxia and tumour aggressiveness in PCa patients. (A) Gene expression analysis of *CA9* in a dataset of PCa samples [25]. *CA9* mRNA levels in prostate samples were compared based on the Gleason score (GS; < 7: *n* = 77; ≥ 7: *n* = 53) of the patient (upper panel) or the tissue origin (bottom panel) (nontumoural tissue (NP): *n* = 29, primary tumours (PT): *n* = 131; metastatic tumours (MET): *n* = 19). The y‐axis represents the Log2‐normalized gene expression. *P*‐value derives from the Mann–Whitney U‐test (upper panel) and the Kruskal–Wallis test (lower panel) (*P*, *P*‐value: *P < 0.05, **P < 0.005). Error bars represent 95% CI. (B, C) Gene expression analysis of the TOP‐5 *DUBs* that positively (B) and negatively (C) correlated with *CA9* in the Taylor *et al*. dataset of PCa samples [25]. *DUB* mRNA levels in prostate were compared based on the Gleason score of the sample (GS; < 7: *n* = 77; ≥ 7: *n* = 53). The y‐axis represents the Log_2_‐normalized gene expression as in A. *P*‐value derives from the Mann–Whitney *U*‐test (*P*, *P*‐value: *P < 0.05, **P < 0.005). Error bars represent 95% CI.

**Fig. S2.** Impact of USP29 expression in PCa cell lines *in vitro* and *in vivo*. (A) Representative western blot analysis of USP29 silencing in PC3 cells overexpressing GFP‐USP29 after treatment with 0.5 μg·mL^−1^ doxycycline (+DOX) for 48 h. Whole‐cell extracts (WCE) were subjected to SDS/PAGE (sodium dodecyl sulphate‐polyacrylamide gel electrophoresis) followed by immunoblotting with the indicated antibodies (*n* = 3 independent experiments). (B) Quantification of colony formation efficiency of PC3 cells silenced with shUSP29.1 relative to control cells. One‐sample *t*‐test (hypothetical value = 1; *P*‐value > 0.05) was used for statistical analysis. Error bars represent SEM (*n* = 3 independent experiments). (C) Relative xenograft tumour growth of PC3 cells silenced with shControl or shUSP29.1 in an inducible manner. Kruskal–Wallis test was used for statistical analysis. Error bars represent 95% CI (*n* = 7–8; *P*‐value > 0.05). (D) Representative western blot analysis of the ectopic expression of USP29 in PC3 cells after treatment with 0.5 μg·mL^−1^ doxycycline (+DOX). Whole‐cell extracts (WCE) were subjected to SDS/PAGE (sodium dodecyl sulphate‐polyacrylamide gel electrophoresis) followed by immunoblotting with the indicated antibodies (*n* = 3 independent experiments). (E) Quantification of foci formation of PC3 cells expressing USP29 in an inducible manner relative to control cells. One‐sample *t*‐test (hypothetical value = 1; *P*‐value > 0.05) was used for statistical analysis. Error bars represent SEM (*n* = 3 independent experiments). (F) Cell cycle analysis in PC3 cells expressing USP29 in an inducible manner. Two‐way ANOVA test was used for statistical analysis. Error bars represent SD (*P*‐value > 0.05) (*n* = 3 independent experiments). (G) Quantification of USP29 overexpressing PC3 cell migration (wound‐healing assay). Data are represented as the migration of USP29 overexpressing relative to control PC3 cells at 24 h. One‐sample *t*‐test (hypothetical value = 1; *P*‐value: **P < 0.005) was used for statistical analysis. Error bars represent SEM (*n* = 4 independent experiments). (H) RT‐qPCR analysis of *ZEB2* (zinc finger E‐box‐binding homeobox 2) and *SNAI2* (zinc finger SNAI2) mRNA expression levels in PC3 cells overexpressing USP29 relative to control PC3 cells. One‐sample *t*‐test (hypothetical value = 1; *P*‐value: *P < 0.05, **P < 0.005) was used for statistical analysis. Error bars represent SEM (*n* = 5 independent experiments).

**Fig. S3.** USP29 regulates HIF‐1α in a noncanonical way. Representative western blot analysis of HEK293T cells silenced with control siRNA or two independent siRNA sequences targeting *USP29 and* transfected with Myc‐HIF‐1α DM^(PP/AA)^ (upper panel) or HA‐USP29 (bottom panel). WCE were subjected to SDS/PAGE followed by immunoblotting with the indicated antibodies (*n* = 3 independent experiments).

**Fig. S4.** Catalytically active USP29 stabilizes HIF‐1α by protecting from proteasome‐mediated degradation. (A) Representative western blot analysis of HEK293T cells transfected with Myc‐HIF‐1α DM^(PP/AA)^ and left untreated or treated with either the proteasome inhibitor MG132 (10 μm), the autophagy inhibitor chloroquine (30 μg·mL^−1^) or both inhibitors together for 6 h. WCE were subjected to SDS/PAGE followed by immunoblotting with the indicated antibodies (*n* = 3 independent experiments). (B) Representative western blot analysis of HEK293T cells co‐transfected with Myc‐HIF‐1α DM^(PP/AA)^ and empty vector or GFP‐USP29, and treated with cycloheximide (CHX, 20 μg·mL^−1^) to inhibit protein synthesis. WCE were collected at the indicated times and subjected to SDS/PAGE followed by immunoblotting with the indicated antibodies (*n* = 3 independent experiments).

**Fig. S5.** USP29 interacts with and deubiquitinates HIF‐α DM^(PP/AA)^. (A) Fluorescence images acquired for HeLa cells transfected with the FRET donor (HIF‐2α DM^(PP/AA)^‐GFP, green channel, left panel) alone or together with the FRET acceptor (mCherry‐USP29; red channel, central panel). The lifetime of the donor was measured, and pseudo‐colour coded fluorescence lifetime images (FLIM channel, right panel) were generated. Data, from 3 independent experiments, is represented as average lifetime of the donor in the absence (*n* = 25) and the presence (*n* = 29) of the FRET acceptor. Unpaired *t*‐test was used for statistical analysis (*P*‐value: ****P < 0.0001). Error bars represent SD. Scale bars are 10 μm long. (B) Representative western blot analysis of HEK293T cells silenced with a control or a siRNA targeting *USP29* and co‐transfected with GFP‐HIF‐1α DM^(PP/AA)^, FLAG‐ubiquitin and either empty vector or siRNA‐resistant HA‐USP29. Cells were treated with the proteasome inhibitor MG132 (10 μm) for 2 h and lysed in the presence of the DUB inhibitor NEM (7 mg·mL^−1^). GFP‐HIF‐1α DM^(PP/AA)^ was pulled down with GFP‐traps® and subjected to stringent washes (8 M urea, 1% SDS). Ubiquitinated and nonubiquitinated GFP‐HIF‐1α DM^(PP/AA)^ protein in the eluate was analysed by immunoblotting with anti‐FLAG and anti‐GFP antibodies, respectively (*n* = 3 independent experiments). (C) Representative western blot analysis of HEK293T cells co‐transfected with GFP‐HIF‐2α DM^(PP/AA)^, FLAG‐ubiquitin and either HA‐USP29 or empty vector. Treatment of cells, pull‐down with GFP‐traps^®^ and subsequent analysis of the ubiquitinated and nonubiquitinated GFP‐HIF‐2α DM^(PP/AA)^ protein in the eluate were performed as in B (*n* = 3 independent experiments).

**Fig. S6.** USP29 targets the C‐terminal part of HIF‐α. (A) Representative western blot analysis of HEK293T cells co‐transfected with Myc‐HIF‐1α^630‐826^ and either empty vector, GFP‐USP29 or GFP‐USP29^C/S^. WCE were prepared and submitted to immunoblotting with the indicated antibodies (*n* = 3 independent experiments). (B) Representative western blot analysis of HEK293T cells co‐transfected with Myc‐HIF‐2α^601‐870^ and either empty vector or GFP‐USP29. WCE were prepared and analysed as in A (*n* = 3 independent experiments). (C) Representative western blot analysis of HEK293T cells co‐transfected with Myc‐HIF‐1α ^630–826^, Myc‐HIF‐1α^630‐713^ or Myc‐HIF‐1α^630‐750^ and either empty vector or GFP‐USP29. WCE were analysed as previously (*n* = 3 independent experiments). (D) Alignment of the lysine‐containing C‐terminal sequence of HIF‐1α from human (H), mouse (M), rat (R), cow (T), xenopus (X) and zebrafish (Z) and human HIF‐2α (H).

## Data Availability

The authors declare that data supporting the findings of this study are available within the paper and its Supporting Information.
