# Supplementary figures and images for: USP29‐regulated noncanonical stabilization of the hypoxia‐inducible factor‐α in aggressive prostate cancer
Source: Mol Oncol. 2026 May 13:10.1002/1878-0261.70268. Online ahead of print. doi: 10.1002/1878-0261.70268 (PMC13398751; doi:10.1002/1878-0261.70268)

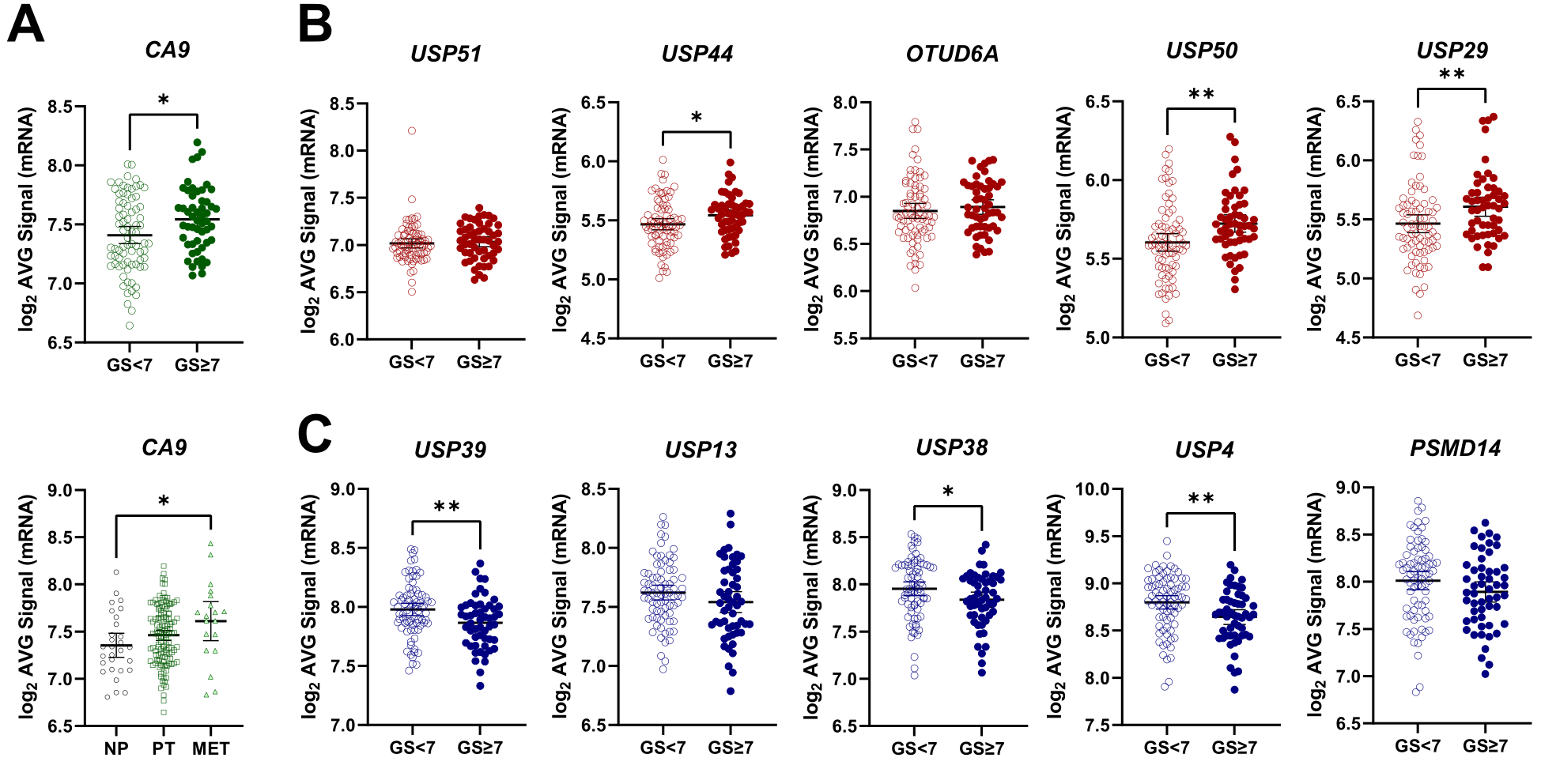

Supplement: Supplementary file 1 — Fig. S1. USP29 correlates with hypoxia and tumour aggressiveness in PCa patients. (A) Gene expression analysis of CA9 in a dataset of PCa samples [25]. CA9 mRNA levels in prostate samples were compared based on the Gleason score (GS; < 7: n = 77; ≥ 7: n = 53) of the patient (upper panel) or the tissue origin (bottom panel) (nontumoural tissue (NP): n = 29, primary tumours (PT): n = 131; metastatic tumours (MET): n = 19). The y‐axis represents the Log2‐normalized gene expression. P‐value derives from the Mann–Whitney U‐test (upper panel) and the Kruskal–Wallis test (lower panel) (P, P‐value: *P < 0.05, **P < 0.005). Error bars represent 95% CI. (B, C) Gene expression analysis of the TOP‐5 DUBs that positively (B) and negatively (C) correlated with CA9 in the Taylor et al. dataset of PCa samples [25]. DUB mRNA levels in prostate were compared based on the Gleason score of the sample (GS; < 7: n = 77; ≥ 7: n = 53). The y‐axis represents the Log2‐normalized gene expression as in A. P‐value derives from the Mann–Whitney U‐test (P, P‐value: *P < 0.05, **P < 0.005). Error bars represent 95% CI. [file MOL2-9999-0-s003.tiff]

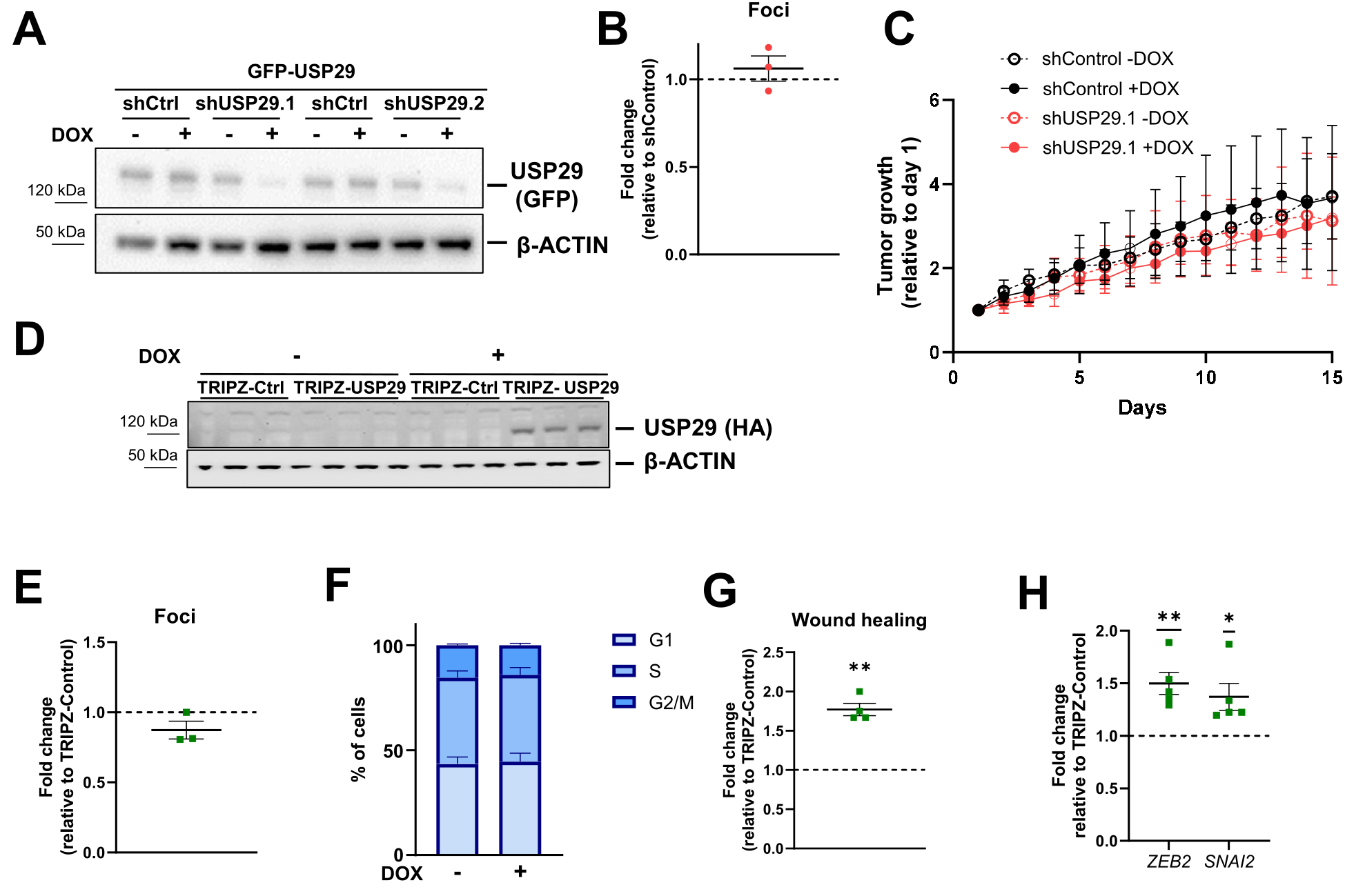

Supplement: Supplementary file 2 — Fig. S2. Impact of USP29 expression in PCa cell lines in vitro and in vivo. (A) Representative western blot analysis of USP29 silencing in PC3 cells overexpressing GFP‐USP29 after treatment with 0.5 μg·mL−1 doxycycline (+DOX) for 48 h. Whole‐cell extracts (WCE) were subjected to SDS/PAGE (sodium dodecyl sulphate‐polyacrylamide gel electrophoresis) followed by immunoblotting with the indicated antibodies (n = 3 independent experiments). (B) Quantification of colony formation efficiency of PC3 cells silenced with shUSP29.1 relative to control cells. One‐sample t‐test (hypothetical value = 1; P‐value > 0.05) was used for statistical analysis. Error bars represent SEM (n = 3 independent experiments). (C) Relative xenograft tumour growth of PC3 cells silenced with shControl or shUSP29.1 in an inducible manner. Kruskal–Wallis test was used for statistical analysis. Error bars represent 95% CI (n = 7–8; P‐value > 0.05). (D) Representative western blot analysis of the ectopic expression of USP29 in PC3 cells after treatment with 0.5 μg·mL−1 doxycycline (+DOX). Whole‐cell extracts (WCE) were subjected to SDS/PAGE (sodium dodecyl sulphate‐polyacrylamide gel electrophoresis) followed by immunoblotting with the indicated antibodies (n = 3 independent experiments). (E) Quantification of foci formation of PC3 cells expressing USP29 in an inducible manner relative to control cells. One‐sample t‐test (hypothetical value = 1; P‐value > 0.05) was used for statistical analysis. Error bars represent SEM (n = 3 independent experiments). (F) Cell cycle analysis in PC3 cells expressing USP29 in an inducible manner. Two‐way ANOVA test was used for statistical analysis. Error bars represent SD (P‐value > 0.05) (n = 3 independent experiments). (G) Quantification of USP29 overexpressing PC3 cell migration (wound‐healing assay). Data are represented as the migration of USP29 overexpressing relative to control PC3 cells at 24 h. One‐sample t‐test (hypothetical value = 1; P‐value: **P < 0.005) w [file MOL2-9999-0-s005.tiff]

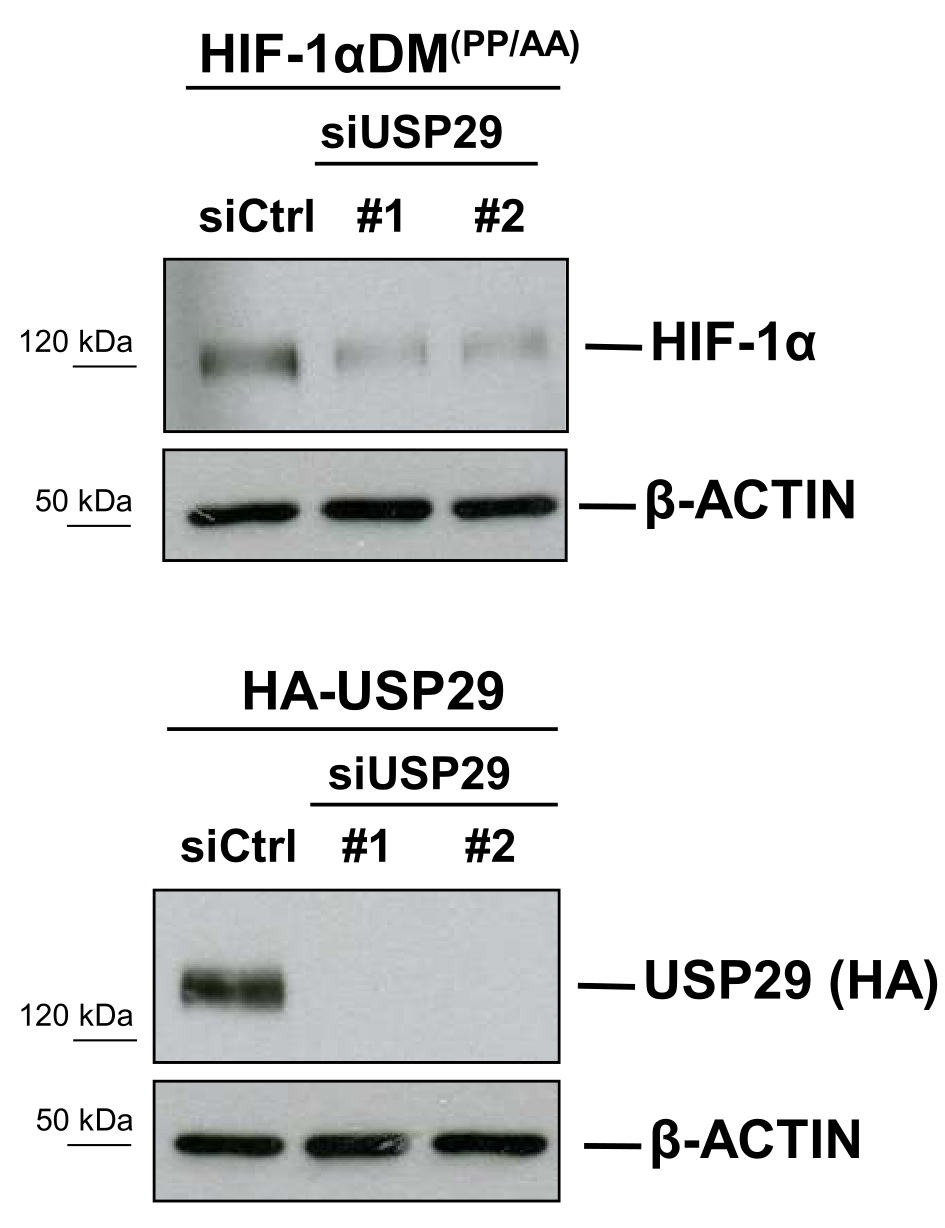

Supplement: Supplementary file 3 — Fig. S3. USP29 regulates HIF‐1α in a noncanonical way. Representative western blot analysis of HEK293T cells silenced with control siRNA or two independent siRNA sequences targeting USP29 and transfected with Myc‐HIF‐1α DM(PP/AA) (upper panel) or HA‐USP29 (bottom panel). WCE were subjected to SDS/PAGE followed by immunoblotting with the indicated antibodies (n = 3 independent experiments). [file MOL2-9999-0-s004.tiff]

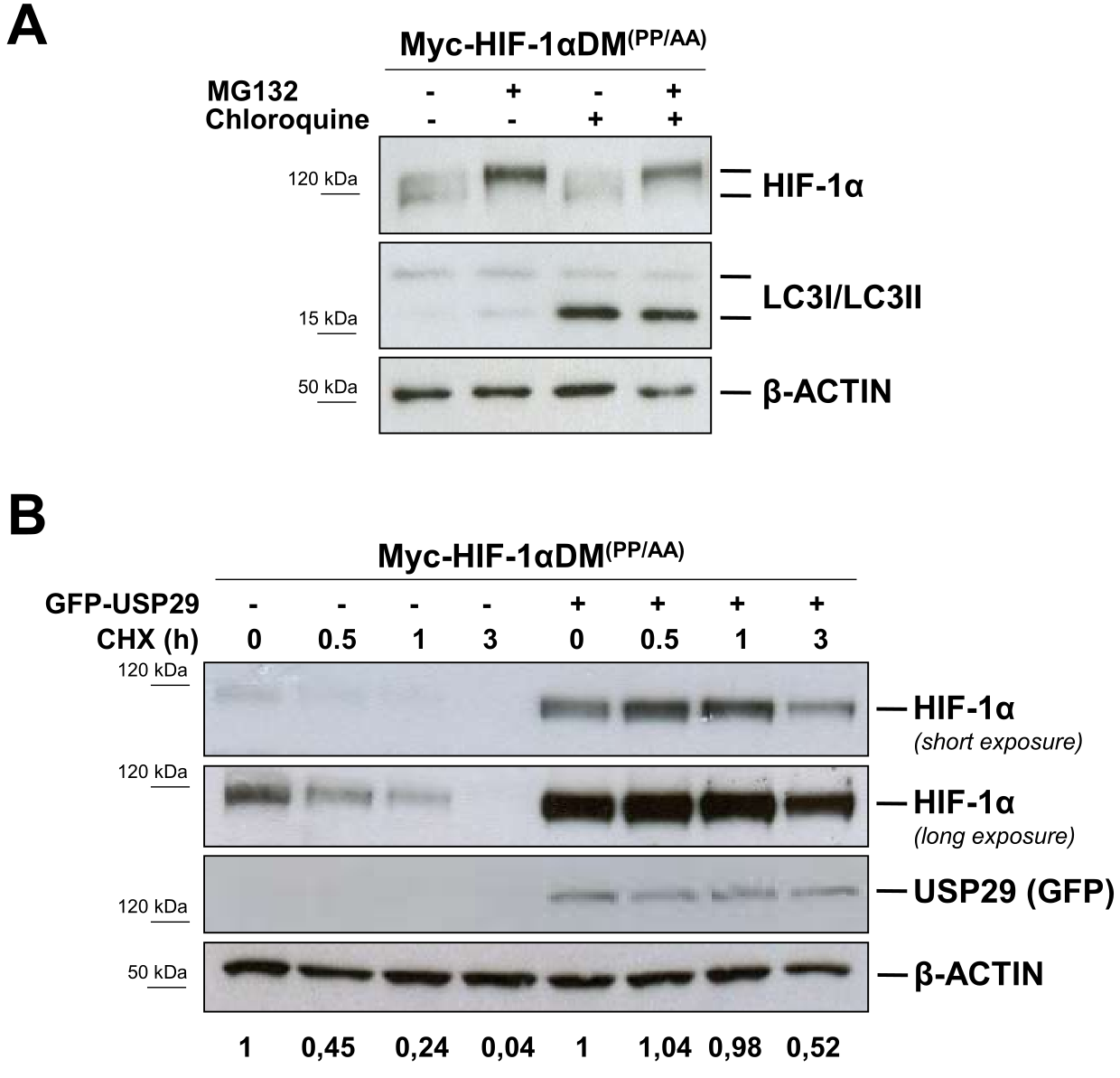

Supplement: Supplementary file 4 — Fig. S4. Catalytically active USP29 stabilizes HIF‐1α by protecting from proteasome‐mediated degradation. (A) Representative western blot analysis of HEK293T cells transfected with Myc‐HIF‐1α DM(PP/AA) and left untreated or treated with either the proteasome inhibitor MG132 (10 μm), the autophagy inhibitor chloroquine (30 μg·mL−1) or both inhibitors together for 6 h. WCE were subjected to SDS/PAGE followed by immunoblotting with the indicated antibodies (n = 3 independent experiments). (B) Representative western blot analysis of HEK293T cells co‐transfected with Myc‐HIF‐1α DM(PP/AA) and empty vector or GFP‐USP29, and treated with cycloheximide (CHX, 20 μg·mL−1) to inhibit protein synthesis. WCE were collected at the indicated times and subjected to SDS/PAGE followed by immunoblotting with the indicated antibodies (n = 3 independent experiments). [file MOL2-9999-0-s001.tiff]

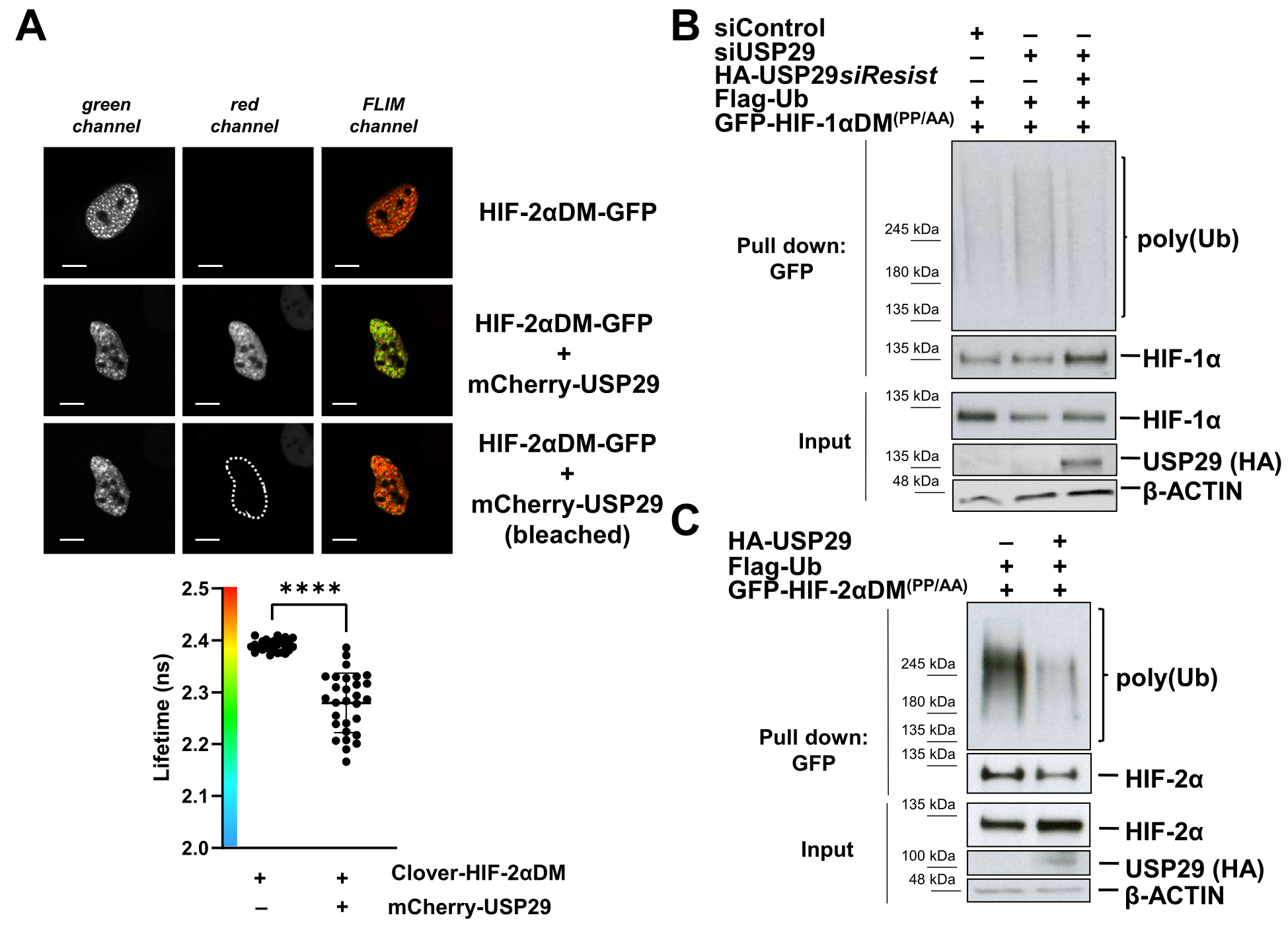

Supplement: Supplementary file 5 — Fig. S5. USP29 interacts with and deubiquitinates HIF‐α DM(PP/AA). (A) Fluorescence images acquired for HeLa cells transfected with the FRET donor (HIF‐2α DM(PP/AA)‐GFP, green channel, left panel) alone or together with the FRET acceptor (mCherry‐USP29; red channel, central panel). The lifetime of the donor was measured, and pseudo‐colour coded fluorescence lifetime images (FLIM channel, right panel) were generated. Data, from 3 independent experiments, is represented as average lifetime of the donor in the absence (n = 25) and the presence (n = 29) of the FRET acceptor. Unpaired t‐test was used for statistical analysis (P‐value: ****P < 0.0001). Error bars represent SD. Scale bars are 10 μm long. (B) Representative western blot analysis of HEK293T cells silenced with a control or a siRNA targeting USP29 and co‐transfected with GFP‐HIF‐1α DM(PP/AA), FLAG‐ubiquitin and either empty vector or siRNA‐resistant HA‐USP29. Cells were treated with the proteasome inhibitor MG132 (10 μm) for 2 h and lysed in the presence of the DUB inhibitor NEM (7 mg·mL−1). GFP‐HIF‐1α DM(PP/AA) was pulled down with GFP‐traps® and subjected to stringent washes (8 M urea, 1% SDS). Ubiquitinated and nonubiquitinated GFP‐HIF‐1α DM(PP/AA) protein in the eluate was analysed by immunoblotting with anti‐FLAG and anti‐GFP antibodies, respectively (n = 3 independent experiments). (C) Representative western blot analysis of HEK293T cells co‐transfected with GFP‐HIF‐2α DM(PP/AA), FLAG‐ubiquitin and either HA‐USP29 or empty vector. Treatment of cells, pull‐down with GFP‐traps® and subsequent analysis of the ubiquitinated and nonubiquitinated GFP‐HIF‐2α DM(PP/AA) protein in the eluate were performed as in B (n = 3 independent experiments). [file MOL2-9999-0-s002.tiff]

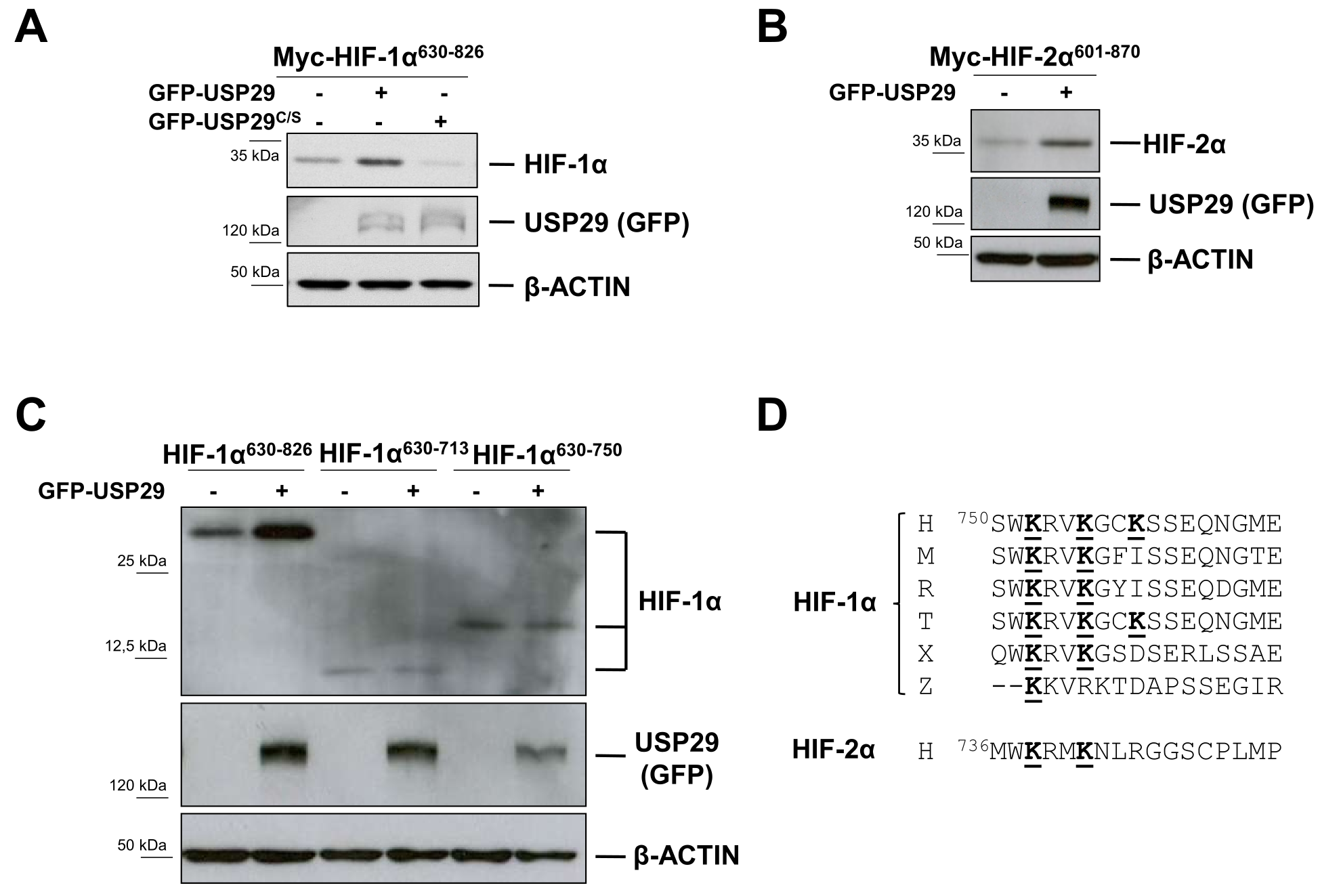

Supplement: Supplementary file 6 — Fig. S6. USP29 targets the C‐terminal part of HIF‐α. (A) Representative western blot analysis of HEK293T cells co‐transfected with Myc‐HIF‐1α630‐826 and either empty vector, GFP‐USP29 or GFP‐USP29C/S. WCE were prepared and submitted to immunoblotting with the indicated antibodies (n = 3 independent experiments). (B) Representative western blot analysis of HEK293T cells co‐transfected with Myc‐HIF‐2α601‐870 and either empty vector or GFP‐USP29. WCE were prepared and analysed as in A (n = 3 independent experiments). (C) Representative western blot analysis of HEK293T cells co‐transfected with Myc‐HIF‐1α 630–826, Myc‐HIF‐1α630‐713 or Myc‐HIF‐1α630‐750 and either empty vector or GFP‐USP29. WCE were analysed as previously (n = 3 independent experiments). (D) Alignment of the lysine‐containing C‐terminal sequence of HIF‐1α from human (H), mouse (M), rat (R), cow (T), xenopus (X) and zebrafish (Z) and human HIF‐2α (H). [file MOL2-9999-0-s006.tiff]
